# An Advanced Communication Skills Workshop Using Standardized Patients for Senior Medical Students

**DOI:** 10.15766/mep_2374-8265.11163

**Published:** 2021-05-27

**Authors:** Jaideep S. Talwalkar, Auguste H. Fortin, Laura J. Morrison, Alan Kliger, David I. Rosenthal, Tanya Murtha, Matthew S. Ellman

**Affiliations:** 1 Associate Professor, Departments of Medicine and Pediatrics, and Director of Clinical Skills, Yale School of Medicine; 2 Professor, Department of Medicine, and Director of Communication Skills Education, Yale School of Medicine; 3 Associate Professor, Department of Medicine, and Director of Hospice and Palliative Medicine Fellowship, Yale School of Medicine; 4 Clinical Professor, Department of Medicine, Yale School of Medicine; 5 Assistant Professor, Department of Medicine, and Director of Capstone Course, Yale School of Medicine; 6 Assistant Professor, Department of Pediatrics (Critical Care Medicine), Columbia University; 7 Professor, Department of Medicine, and Director of Medical Student Palliative and End-of-Life Care Education, Yale School of Medicine

**Keywords:** Communication Skills, Clinical Skills Assessment/OSCEs, Standardized Patient, Peer-Assisted Learning, Strong Emotion, Goals of Care, Medical Errors, End of Life/Palliative Care

## Abstract

**Introduction:**

Medical students often lack training in advanced communication skills encompassing emotionally fraught situations and those in which an intense emotional response is expected. Such skills are required for clinical situations encountered during residency. We created and evaluated an advanced communication skills workshop (ACSW) using standardized patients for senior medical students. The workshop emphasized communication skills for four scenarios—strong emotion, goals of care, medical error, and palliative care assessment—and utilized formative peer assessment and feedback.

**Methods:**

We created the four ACSW cases with case-specific communication behavior checklists and a common modified Master Interview Rating Scale in a Capstone Course for senior medical students. In groups of three, students rotated through three of four stations. Each student conducted one of the interviews while the other two completed the checklists and provided verbal feedback. We performed one-way analyses of variance on Likert responses and content analysis on open responses on a post-ACSW survey.

**Results:**

Ninety-one students completed the ACSW and survey. Students assigned high value to all four ACSW student roles: interviewer, observer, feedback recipient, and feedback provider. Students rated the experience above average to excellent on nearly all survey items. Open-response themes included “liked the opportunity to give or receive peer feedback” (46%) and “found the checklists helpful” (45%).

**Discussion:**

Feasible and well received by senior medical students, our ACSW offers an opportunity to practice and observe advanced communication skills and peer feedback. A peer-assisted, formative learning model, the ACSW efficiently addresses a key aspect of residency preparation.

## Educational Objectives

By the end of this activity, learners will be able to:

1. Practice strategies for managing difficult encounters with patients who display strong emotions.
2. Discuss goals of care and resuscitation preferences with a family member of a critically ill patient.
3. Demonstrate a model for disclosing a medical error to a patient.
4. Take a palliative care history of a patient with no remaining curative treatment options.
5. Deliver checklist-driven feedback to a peer after observing a standardized patient encounter.
6. Receive feedback from peers after communicating with a standardized patient under specific clinical circumstances.

## Introduction

The acquisition of clinical skills, the essential elements of doctoring that students will bring to patient encounters throughout their careers, is a core aspect of medical training.^1^ Communication with patients and families is a fundamental clinical skill,^2^ and communication skills are used by patients and families as well as certifying and licensing boards as measures of physician competence.^3,4^ Observed structured clinical examinations (OSCEs) using standardized patients (SPs) are widely utilized to both teach and assess basic communication skills during medical school.^5–7^ However, when these students become residents, they are expected to manage clinical situations requiring more advanced communication skills,^1^ in which clinicians must navigate emotionally fraught circumstances and in which an intense emotional response from patients or families can be expected.^8^

During medical school, students rarely have the opportunity to lead conversations requiring advanced communication skills. This has led some educators to develop OSCEs^1^ for students to show how one might interact with a real patient.^9^ Descriptions of such OSCEs have focused on important communication competencies such as motivational interviewing,^10,11^ delivering serious news,^10,12^ and other specific clinical circumstances.^13,14^ More OSCEs that address advanced communication skills have targeted postgraduate trainees (i.e., residents, fellows) on topics such as error disclosure,^15,16^ goals of care,^15^ and specialty-specific content.^16–18^ However, none of these OSCEs have utilized a pure peer-feedback model, and the number and breadth of cases and descriptions are still limited compared to OSCEs on more basic content.^5^

During postgraduate training, residents rarely receive direct observation and feedback related to their advanced communication skills.^19^ This is despite longstanding recognition that emphasis on clinical skills training during residency is essential for ongoing maturation of the clinician at the bedside.^20^ Given the amount of time residents spend together, formal peer-feedback mechanisms have been incorporated into postgraduate training.^21,22^ For feedback to be most useful, deliberate instruction in giving and receiving feedback is valuable.^23^

Prior to 2017, the curriculum for senior medical students (MS4s) at our school contained didactic content and role-play exercises to teach advanced communication skills topics but only a single OSCE station on palliative care assessment.^24^ Given the success of the existing OSCE station, we sought to expand the advanced communication skills curriculum by developing a multistation formative advanced communication skills workshop (ACSW) with SPs to address strong emotion (anger), goals of care, medical error, and palliative care assessment. We have previously demonstrated that MS4s performed well during the ACSW and that peer assessment was concordant with assessment done by SPs and faculty.^8^ Here, we describe the development of the ACSW and evaluate student satisfaction with the ACSW, specifically as related to the student-only assessment model in which students had the opportunity to enhance their skills in giving and receiving peer feedback. Unlike previously published OSCEs related to advanced communication skills, the ACSW solely relies on a peer-feedback model with medical students.

## Methods

### Curricular Context

We built the ACSW into the required Capstone Course held prior to graduation for MS4s at our institution. During the 3-week Capstone Course, students reflected on knowledge acquired during medical school and learned additional skills that would be required during residency. Through lectures and workshops, students discussed high-yield topics especially relevant for residency (e.g., interpretation of electrocardiograms, anaphylaxis management, wellness practices, personal finance). All activities in the course were formative and ungraded. Earlier in the curriculum, students had completed OSCEs on more basic content (e.g., patient-centered interviewing, delivering serious news, problem-focused visits). There was no formal instruction or structured practice in peer feedback. As part of a multiyear redesign of the entire curriculum in 2017, the Capstone Course was revised, providing opportunity for novel teaching strategies. The ACSW as described here took place during the first year of the revised course in 2017.

### Pilot Phase

In 2016, we convened a 10-person working group consisting of members of the Clinical Skills Instruction team, Standardized Patient Program, Teaching and Learning Center,^25^ and Capstone Course team. The group reviewed existing content related to advanced communication skills in the Capstone Course and elsewhere in the curriculum with the goal of developing a multistation OSCE. The working group considered three possible assessment models: direct assessment by faculty, peer assessment, and assessment by the SPs. We presented these models to senior students, who were unenthusiastic about direct faculty observation on challenging new skills so close to graduation but felt that both peer and SP assessment would be well received. Given that direct faculty observation for all OSCE stations would also be resource intensive, we piloted peer and SP assessment utilizing an assessment checklist previously designed for the palliative care station.^24^

We recruited six senior student volunteers who had not previously completed the palliative care case for a single-station pilot in May 2016. Two groups of three MS4s and an SP who had extensive experience in giving feedback and was trained on the palliative care assessment script participated. Students received a brief overview of session logistics and were told to provide behavior-based feedback relying on the assessment checklist. In each trio, student A performed a 20-minute interview of the SP while students B and C observed and completed the assessment checklist. Upon completion of the interview, students B and C gave checklist-based feedback to student A. Faculty in the working group observed live video streams of each of the two sessions, which were also recorded. Upon completion of the exercise, faculty debriefed with the students and the SP.

During the debriefing of the pilot, students universally reported finding the experience valuable. Students who had interviewed the SP appreciated the opportunity to practice a higher-level communication skill and found feedback from classmates to be helpful. Students who had observed reported learning from their classmate's performance and from the checklist itself. Students reported that they took the exercise seriously out of recognition of the importance of the skills being addressed and the reality of internship on the horizon. One student said, “I'd rather practice this now than do it for the first time as an intern in the Emergency Department.” Students commented that they preferred not having faculty in the room as it made them less self-conscious about both the interview and giving feedback: “If the expert is in the room, I wouldn't feel like I have anything useful to add.” Overall students were enthusiastic about the prospect of such an exercise conducted on a broader scale, covering more topics.

The SP reported that the feedback the students had provided mirrored closely what he would have said. He was impressed with the depth and accuracy of student feedback. Similarly, faculty were impressed with the quality of the student interviews and with the feedback provided to interviewers, which was constructive, specific, and actionable.

### ACSW Case and Checklist Design

Given the success of the pilot, the working group adopted a peer-assessment model and developed a final list of scenarios for the ACSW: strong emotion (anger), goals of care, medical error, and palliative care assessment. We invited faculty experts in each content area to design the cases and preliminary checklists for the three remaining topics. The cases and checklists underwent multiple rounds of revision between the faculty experts and the working group, with final development of communication behavior checklists (CBCs). Two members of the working group (Jaideep S. Talwalkar and Matthew S. Ellman) independently ranked the 28-items on the Master Interview Rating Scale (MIRS) based on applicability to all stations and lack of redundancy with items on the CBCs. The MIRS is a widely used instrument measuring patient-centered communication skills.^26,27^ After reconciling the rank lists and reviewing the selections with the faculty experts and working group, we adopted a nine-item modified MIRS (mMIRS) for use across the four OSCE stations. Through this process, the working group modified the existing palliative care case and checklist to ensure consistency in style and format with the other stations.

We recruited 12 SPs from our Standardized Patient Program to participate in the OSCE, three for each of the four cases. SPs studied the cases and then participated in a separate 2-hour training session for each case. Training sessions were run by a member of the Standardized Patient Program plus a faculty content expert. During training, SPs reviewed medical details of the case, discussed emotional context, and practiced playing their roles. SPs also received training on using the CBC as part of a separate study related to this project; SP assessments were not shared with students and were only performed to assess concordance with peer assessment.^8^

### Workshop Logistics

In the days before the ACSW, didactic presentations and demonstrations on various advanced communication skills topics were included in the Capstone Course. These sessions addressed the topic for each scenario in the ACSW, among other content. Students also had a session on giving and receiving behavior-based peer feedback, as well as on the logistics of the ACSW, to minimize announcements during the workshop. Students learned about the objectives and structure of the ACSW but not the specific communication topics in the OSCE. During the ACSW itself, 12 students at a time rotated in a predetermined order through three of the four stations in randomly assigned groups of three (Appendix A). Similar to the pilot, student A performed a 20-minute interview of the SP while students B and C observed. Prior to entering the room, student A reviewed the case background materials, while students B and C received the case vignette along with the CBC and mMIRS (Appendices B–E). Upon completion of the interview, the SP left the room to take a break, and student A was given 2 minutes to complete the CBC (i.e., self-assessment) while students B and C completed the CBC and mMIRS (i.e., peer assessment). Students B and C then facilitated an 8-minute feedback session with student A. The group next moved through two more stations, with students rotating roles such that each student interviewed once and observed twice (Appendix F). After completion of three stations, students filled out an anonymous six-item survey about the workshop consisting of five items rated on a 5-point Likert scale (1 = *Poor,* 2 = *Below Average,* 3 = *Average,* 4 = *Above Average,* 5 = *Excellent*) and an additional open-response prompt (Appendix G). The 12 students in each wave then attended a 30-minute debriefing session with faculty involved in ACSW development; during the debriefing, they reviewed the cases and checklists and discussed the workshop in general (Appendix H).

Two staff and one faculty were always present to guide students and SPs, answer questions, handle paperwork (i.e., scripts, checklists), check in with SPs informally, and run the debriefing session. Immediately following the session, apart from the educational innovation, Standardized Patient Program faculty reached out to SPs by email to offer additional debriefing if needed. In the weeks following the ACSW, the working group, which included an SP from the ACSW, reconvened to debrief and plan for the following year.

The $3,000 cost of the ACSW was entirely related to paying SP wages. This figure does not include the time of salaried faculty and staff or the overhead costs of the facility (e.g., practice rooms).

### Analysis

We performed one-way analyses of variance on the Likert responses to the post-OSCE survey. Qualitative analysis was done on the open responses by performing content analysis to identify repetitive themes. Text was coded using these themes, and frequencies were calculated. Statistical analysis was performed using Social Science Statistics and Microsoft Excel 2016.

## Results

All 91 MS4s participated in the ACSW. Completion of the post-OSCE survey indicated consent to utilize the survey data in the curriculum evaluation, and surveys were completed by 100% of participants. On the Likert-scale items, students assigned high value to their four session roles: interviewer and observer during the SP interviews and recipient and provider during the feedback sessions. Additionally, students valued peer feedback and the self-assessment CBCs. Responses were in the above average to excellent range for the all-case averages on all five Likert items and for all but one of the relevant items for the individual cases (Table 1).

**Table 1.**
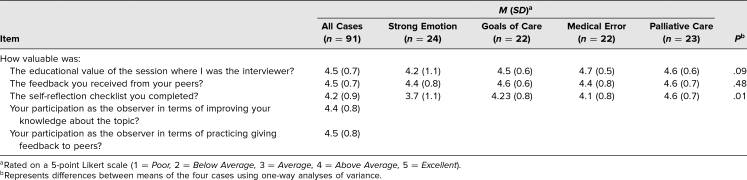
Average Responses to Likert-Scale Items on Post-OSCE Survey (*n* = 91)

Mean satisfaction scores related to educational value and self-reflection on the strong emotion case were lower than for the other cases; the difference in self-reflection scores across the cases was statistically significant (*p* = .01; see Table 1). The strong emotion case elicited the only responses to the entire survey indicating a poor experience, and standard deviations were wider.

Comments on the open-response prompt were submitted by 69 students (76%). Content analysis identified 11 distinct themes plus two categories for nonspecific positive and negative comments. Responses contained between one and four themes each. Table 2 lists the themes along with representative comments. Notably, 32 respondents (46%) liked the opportunity to give or receive peer feedback, 31 (45%) found the checklists helpful, and eight (12%) valued the opportunity to self-assess or reflect.

**Table 2.**
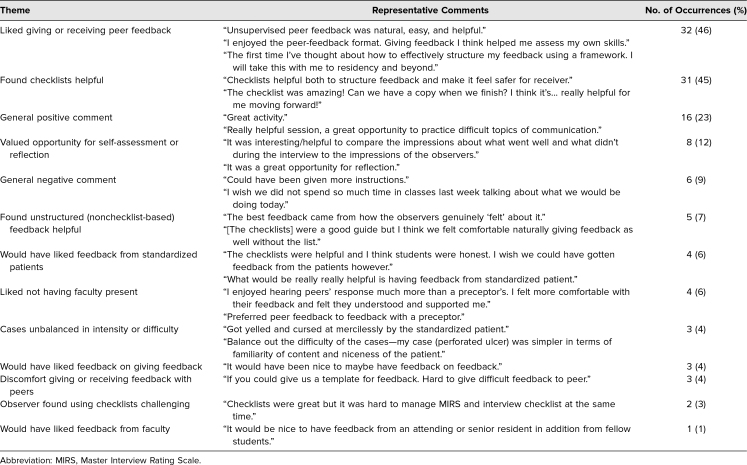
Themes on Qualitative Analysis (*n* = 69)

In the working group debriefing session, staff and faculty team members reviewed survey data, made observations, and proposed ideas to improve and simplify the workshop in future iterations. Observations made during the working group debrief included the following:

• Students exhibited professional behavior and appeared to take the session quite seriously.
• Students were engaged during the debriefing session and appreciated the chance to reflect on their experiences on clerkship compared to the workshop.
• Some students seemed upset by the strong emotion case and felt it was unfair. The actors’ reactions could be heard clearly through the walls.
• Faculty member was not needed in between debriefing sessions.
• The workshop stayed on schedule.
• Some students seemed confused about the flow of the workshop despite the reminders.
• Keeping paper checklists organized was a full-time job for one staff member.

## Discussion

We developed a four-station ACSW for senior medical students that relied entirely on a peer-feedback model for formative assessment. Throughout the workshop, students practiced skills that are key elements of residency preparation.^1^ In addition to practicing and observing OSCE stations covering strong emotion, goals of care, medical error, and palliative care assessment, students practiced self-assessment and giving and receiving behavior-based peer feedback.

The ACSW was efficient to administer and well received by senior medical students. Likert responses suggested that students found value in all of their session roles: interviewer and observer during the SP interviews and recipient and provider during the feedback sessions. We found that the ACSW was an efficient way to foster important advanced communication skills topics in terms of cost, faculty utilization, and curricular time.

The ACSW required the extensive planning and orchestration typical of OSCEs,^7,28^ but despite its complexity, the session remained cost neutral compared to our existing OSCE on palliative care, which had relied on SP and expert-rater assessment.^24^ By utilizing a student-only assessment model, we were able to trim resources that had added to costs to the original case. Since the ACSW was purely formative, we felt that high-level quality assurance with extensively trained raters was unnecessary. Instead, we put all of our financial resources towards training SPs to portray the medically and emotionally complex roles we developed. Previous research demonstrated that rating abilities of SPs can diminish as the cognitive demands of their roles increase.^29^ With training, SPs can achieve high reliability in rating during complex scenarios,^24^ but we determined that the investment of time and resources to achieve this was unnecessary to meet our educational objectives. In a related study, we previously reported sufficient concordance among different types of raters to support a student-only assessment model for our low-stakes, formative ACSW.^8^

Additionally, the ACSW was efficient in terms of faculty utilization. Faculty were heavily involved in the curriculum development phase but played a minimal role during the workshop itself. Thus, our design relied not on feedback from faculty or SPs but on self-assessment and peer feedback. This allowed us to avoid the challenge of recruiting additional faculty. Students recognized the benefits of giving and receiving feedback, learning from the checklists while in the observer role, and having dedicated time for self-reflection. We were not surprised that the model was well received by students based on our pilot and previous work describing the benefits of peer-assisted learning^30,31^ and the quality of our behaviorally based checklists that had undergone multiple rounds of revision.

The ACSW did not utilize additional curricular time because it replaced existing content using a more interactive format. While the workshop itself took 14 hours for the entire class of 91 students, each individual student participated for only 2 hours, creating opportunities for other small-group activities during downtime. The time to get the entire class through the ACSW could be reduced by running more concurrent stations, which would involve training more SPs for each case. We chose not to include the preworkshop content in the tally of curricular hours or as part of the ACSW itself because that content was spread over time and not directly linked to the cases. Others wishing to adopt a similar approach would need to determine how to address the content in the curriculum prior to the ACSW to meet their students’ needs.

While all cases were generally well received, there was more variability in response on the strong emotion case—in which the patient was very angry—than on the other three OSCE stations. Student comments and the faculty working group noted that the cases were unbalanced in their intensity, and this strong emotion case was particularly charged. The script was written so that the patient would “be livid and respond with anger” early in the case, regardless of what the student had done beforehand. The key task for the interviewing student was to recognize the emotion and respond with empathy, rather than becoming defensive or responding with a fact-based discussion, which were skills presented and demonstrated in the preceding didactic session. When clinicians and patients have misaligned expectations, both parties may experience emotions including anger and dissatisfaction.^32^ Therefore, it was critical to include an exploration of student discomfort in the faculty debriefing session to help students handle difficult interactions. Similarly, while not part of the curricular evaluation, we recognized the possible emotional toll of challenging roles on the SPs; we limited the amount of time in role and offered breaks and debriefing opportunities.^7^

We identified several limitations through curriculum evaluation and made changes in the second year of administering the ACSW in response to lessons learned during the first year of implementation. With instructions, vignettes, and checklists for four cases, the amount of paperwork was a burden on program staff. In subsequent sessions, we have uploaded all material to the online student course management system just before the start of the session, and students have used their school-issued tablets to navigate the instructions (Appendix F), vignettes, and checklists. By moving away from paper, we have been able to reduce the amount of administrative support during the workshop. Since some students were confused about the flow of the ACSW and other students perceived too much or too little in-class instruction before the session, we have replaced the in-class ACSW orientation with a video walk-through for students to view before the session. We anticipate this will be well-received since we have successfully used such technology to engage our learners elsewhere in the curriculum.^33^ Additionally, we have pooled best practices of faculty who ran the debriefing session and developed a debriefing guide (Appendix H). Finally, educators may note that the vignette for the strong emotion case was written with language that could lead students to go into the encounter with a negative impression of the patient. Educators wishing to prime students with a more patient-centered mindset can consider substituting two statements in the vignette. “The patient has not been easy to care for from the outset” can be replaced with “The patient has met with challenges in care delivery from the outset,” and “has brought a long list of issues for you to deal with” can be replaced with “has brought a long list of concerns for you to address.”

We have instituted a system to address variability in emotional intensity across cases and SPs. We now offer SPs the opportunity to have sessions recorded (with permission from the students) to hone their performances and learn from those of their peers. Regarding the strong emotion case, we have asked SPs to moderate the extent of their expressed anger. While the case still calls for the patient to be livid, SPs are coached to express their emotion at a lower volume and modulate their anger in response to expressions of empathy, with the hope that no student will perceive emotional intensity that overshadows the educational value of the workshop.

The innovation described was conducted with a single class of fourth-year students at an institution with a well-established Standardized Patient Program.^7^ While the use of OSCEs is common in medical education, generalizability of the educational approach to other programs would hinge on the availability of SPs with similar skill in portraying intense emotional responses and of instructors to train and debrief with them. Additionally, the ACSW was designed to be a formative activity within an ungraded course, which may limit applicability to programs with different assessment structures.

Given the success of the four-station ACSW, we plan to expand the workshop to include additional stations on delivering serious news over the telephone and death notification. Our students have found the ACSW valuable, and we encourage other institutions to adopt the use of practicing advanced communication skills in simulation with SPs for senior medical students.

## Appendices

Schedule & Logistics.xlsxStrong Emotion Case Materials.docxGoals of Care Case Materials.docxError Disclosure Case Materials.docxPalliative Care Case Materials.docxStudent Instructions.docxPostsession Survey.docxFaculty Debrief Guide.docx
*All appendices are peer reviewed as integral parts of the Original Publication.*
