# Supplementary material for: An Advanced Communication Skills Workshop Using Standardized Patients for Senior Medical Students
Source: MedEdPORTAL. 2021 May 27;17:11163. doi: 10.15766/mep_2374-8265.11163 (PMC8155077; doi:10.15766/mep_2374-8265.11163)
Supplement: Supplementary file 1 — Schedule & Logistics.xlsxStrong Emotion Case Materials.docxGoals of Care Case Materials.docxError Disclosure Case Materials.docxPalliative Care Case Materials.docxStudent Instructions.docxPostsession Survey.docxFaculty Debrief Guide.docx [file mep_2374-8265.11163-s001.zip › B. Strong Emotion Case Materials.docx]

**Material for Student Interviewer**

**Setting:** You are a surgical resident. You are about to meet with a patient who has just returned to the hospital room following an operation that you performed with the attending.

**Opening Scenario (read this carefully before entering the room):**

The patient is a ___-year-old man/woman with Crohn’s disease on the surgery service. You have just done a partial small bowel resection (for strictures) which was unexpectedly complicated, and required a loop ileostomy rather than the expected end-to-end anastomosis. During pre-operative planning, you and your attending had told the patient there was a small chance of this happening but you did not expect it.

The patient has not been easy to care for from the outset. You have met him/her a couple of times before, and the patient has chastised you for running even 15 minutes late. The patient has a reputation around the office for being “difficult,” and the secretary has complained to you about the number of calls from this patient about relatively minor concerns. The two times that you’ve seen the patient in clinic, he/she brought a long list of issues for you to deal with, including many that had nothing to do with the Crohn’s disease and the upcoming surgery, but were more primary care type of concerns.

If the patient asks about whether ileostomy could be reversed, you can say that it’s possible but would take at least 3-6 months (or possibly longer) in order to allow any adhesions to soften and decrease risk of injury such as enterotomies.

Vitals: afebrile, P 82, R 12, BP 140/82

**Student Tasks:**

Give the news about the ileostomy to the patient. You enter the hospital room to tell your patient this news, knowing the patient will be unhappy. You have up to 20 minutes with the patient. You do not need to do a physical exam.

**Self-assessment Communication Behavior Checklist for Student Interviewer**

**Complete the following checklist based on the interview you just performed:**

| 1. I elicited the patient's understanding of what's going on before breaking the ileostomy news. | ( ) Yes | ( ) Partial | ( ) No |
| --- | --- | --- | --- |
| 2. I provided a brief explanation about the ileostomy, and then paused to allow time for patient to react emotionally. | ( ) Yes | ( ) Partial | ( ) No |
| 3. I responded to patient emotion with verbal empathic statements/appropriate body language. | ( ) Yes | ( ) Partial | ( ) No |
| 4. I explored what having an ileostomy means in the context of this patient's life. | ( ) Yes | ( ) Partial | ( ) No |
| 5. I avoided detailed fact-based discussion until after emotion-seeking had occurred. | ( ) Yes | ( ) Partial | ( ) No |

**Material for Student Observers (also suitable for other assessors – SPs, faculty, etc.)**

**Tasks for Student Observers:**  You will observe a classmate having a conversation with a patient. Complete the history checklist on the next page as you observe your classmate and be prepared to provide feedback at the end of the 20 minute interview. Your classmate has been given the following instructions.

**Setting:** You are a surgical resident. You are about to meet with a patient who has just returned to the hospital room following an operation that you performed with the attending.

**Opening Scenario (read this carefully before entering the room):**

The patient is a ___-year-old man/woman with Crohn’s disease on the surgery service. You have just done a partial small bowel resection (for strictures) which was unexpectedly complicated, and required a loop ileostomy rather than the expected end-to-end anastomosis. During pre-operative planning, you and your attending had told the patient there was a small chance of this happening but you did not expect it.

The patient has not been easy to care for from the outset. You have met him/her a couple of times before, and the patient has chastised you for running even 15 minutes late. The patient has a reputation around the office for being difficult, and the secretary has complained to you about the number of calls from this patient about relatively minor concerns. The two times that you’ve seen the patient in clinic, he/she brought a long list of issues for you to deal with, including many that had nothing to do with the Crohn’s disease and the upcoming surgery, but were more primary care type of concerns.

If the patient asks about whether ileostomy could be reversed, you can say that it’s possible but would take at least 3-6 months (or possibly longer) in order to allow any adhesions to soften and decrease risk of injury such as enterotomies.

Vitals: afebrile, P 82, R 12, BP 140/82

**Student Tasks:**

Give the news about the ileostomy to the patient. You enter the hospital room to tell your patient this news, knowing the patient will be unhappy. You have up to 20 minutes with the patient. You do not need to do a physical exam.

**Communication Behavior Checklist for Student Observer**

**(also suitable for other assessors – SPs, faculty, etc.)**

**Complete the following checklist during the interview as you observe:**

| 1. Elicits patient's understanding of what's going on before breaking the ileostomy news. | ( ) Yes | ( ) Partial | ( ) No |
| --- | --- | --- | --- |
| 2. Provides brief explanation of statement of ileostomy, and then pauses to allow time for patient to react emotionally. | ( ) Yes | ( ) Partial | ( ) No |
| 3. Responds to patient emotion with verbal empathic statements/appropriate body language. | ( ) Yes | ( ) Partial | ( ) No |
| 4. Explores what having an ileostomy means in the context of this patient's life. | ( ) Yes | ( ) Partial | ( ) No |
| 5. Avoids detailed fact-based discussion until after emotion-seeking has occurred. | ( ) Yes | ( ) Partial | ( ) No |

**Modified Master Interview Rating Scale (MIRS) for Student Observer**

**(also suitable for other assessors – SPs, faculty, etc.)**

The full MIRS can be found in Supplement 1 of Baldwin JD, Cox J, Wu ZH, Kenny A, Angus S. Delivery and Measurement of High-Value Care in Standardized Patient Encounters. Journal of Graduate Medical Education. 2017;9:645-449. [https://doi.org/10.4300/JGME-D-17-00016.1](https://nam12.safelinks.protection.outlook.com/?url=https%3A%2F%2Fdoi.org%2F10.4300%2FJGME-D-17-00016.1&data=04%7C01%7Cjaideep.talwalkar%40yale.edu%7Cb3f1b75239754625b97308d8cecb8002%7Cdd8cbebb21394df8b4114e3e87abeb5c%7C0%7C0%7C637486720727321375%7CUnknown%7CTWFpbGZsb3d8eyJWIjoiMC4wLjAwMDAiLCJQIjoiV2luMzIiLCJBTiI6Ik1haWwiLCJXVCI6Mn0%3D%7C3000&sdata=JvGyuedfM5vMJOVISVQZh1SaegcnYwZzlZCp2cdfZbw%3D&reserved=0)

Items from MIRS used by Student Observers:

1. Opening

12. Questioning Skills – Lack of Jargon

14. Interactive Techniques

15. Verbal Faciliation Skills

16. Non-Verbal Facilitation Skills

17. Empathy and Acknowledging Patient Cues

22. Patient’s Education & Understanding

27. Encouragement of Questions

28. Closure

**Case script for Standardized Patient**

**Standardized Patient Name:** use your regular character’s name
**Actor:**

**Age:** 30-55
**Episode:** Strong Emotion
**Workshop:** Advanced Communication Skills, Fourth Year Capstone Course
______________________________________________________________________________

You are a patient in a hospital room. You just returned from the post-surgery recovery area, having undergone a small bowel surgery (“partial small bowel resection”) for Crohn’s disease-related strictures a few hours ago. You have recently come out of anesthesia but are fully awake. Your surgical resident (the assistant to the attending surgeon) is about to walk in and break the bad news that he/she had to put in an “ileostomy,” rather than the “end-to-end anastomosis” that you expected – and were told was the plan. You have a good handle on what these terms mean. Although weak from surgery and in pain, you will be livid and respond with anger. You cannot believe how your doctor “lied to me.” He/she “promised me that I wouldn’t need a bag.” You have called the office many times in recent weeks to go over the plan and to ask questions and your understanding was that it was highly unlikely that “I’d end up with a bag.” Your doctor only called back a few times, otherwise you always had to speak with the nurse, and you wonder if all of your concerns were made clear.

You don’t know how you will function. You feel that your social life will be ruined. You just began dating again after a messy divorce three years ago. You are afraid this new person won’t want to date you any longer – “who would want to date someone with a bag?!” You are afraid of the unknown and you are also enraged and overwhelmed. Communicate your emotions to the doctor and see if he/she responds in a way that makes you feel heard, cared for, and understood. If so, *you should feel your anger give way to another emotion*, perhaps sadness or fear. See if your doctor can empathize with these emotions as well.

Background: Fill in background details that come up as you see fit, along with a history of Crohn’s disease for at least ten years. For years you have dealt with abdominal pain, diarrhea (sometimes bloody), and weight loss. This was a huge challenge in your previous marriage. You have been on multiple drugs for this over the years including methotrexate, infliximab, and others. You have had several bowel obstructions and strictures and recently you and your doctors decided that surgery was the only option.

***MedEdPORTAL* Standardized Patient Case Development Tool**

Date: January 26, 2021

Primary Case Author: Yale School of Medicine Advanced Communication Skills Workshop group

Secondary Case Author: Not applicable

Standardized Patient Educator: Not applicable

Name of Case: Strong Emotion

Name of educational and or assessment activity: Advanced Communication Skills Workshop

Patient Name: Character’s regular name

Chief Concern: Post-operative visit

Most likely Diagnosis and Differential with rationale from history and/or physical exam: Not applicable

Challenge question:

You are a surgical resident. You are about to meet with a patient who has just returned to the hospital room following an operation that you performed with the attending.

The patient is a (insert actor’s age)-year-old man/woman with Crohn’s disease on the surgery service. You have just done a partial small bowel resection (for strictures) which was unexpectedly complicated, and required a loop ileostomy rather than the expected end-to-end anastomosis. During pre-operative planning, you and your attending had told the patient there was a small chance of this happening but you did not expect it.

The patient has not been easy to care for from the outset. You have met him/her a couple of times before, and the patient has chastised you for running even 15 minutes late. The patient has a reputation around the office for being difficult, and the secretary has complained to you about the number of calls from this patient about relatively minor concerns. The two times that you’ve seen the patient in clinic, he/she brought a long list of issues for you to deal with, including many that had nothing to do with the Crohn’s disease and the upcoming surgery, but were more primary care type of concerns.

If the patient asks about whether ileostomy could be reversed, you can say that it’s possible but would take at least 3-6 months (or possibly longer) in order to allow any adhesions to soften and decrease risk of injury such as enterotomies.

Vitals: afebrile, P 82, R 12, BP 140/82

Your task is to give the news about the ileostomy to the patient. You enter the hospital room to tell your patient this news, knowing the patient will be unhappy. You have up to 20 minutes with the patient. You do not need to do a physical exam.

Domains: Check all that apply

- Professionalism
- Communication and Interpersonal skills
- Medical History
- Physical exam
- Shared Decision Making
- Patient Education
- Clinical Reasoning
- Documentation
- Handoff
- Presentation
- Other:

Type and level of learner: Senior medical student

Case Objectives: please list specific objectives for each of the domains you have checked above:

1. Deliver difficult news to a patient regarding an unexpected surgical outcome

2. Respond to strong emotions exhibited by a patient with verbal empathic statements

3. Respond to strong emotions exhibited by a patient with appropriate body language

| SETTING: outpatient, in patient, ED, home, nursing home, rehab, group etc. | Hospital room, surgical unit |
| --- | --- |
| PATIENT PROFILE: Information about the “patient” that helps select an SP and helps the learner get an understanding of them as a person. SP will know more information about the patient than learner will ever ask but allows SP to portray a fully developed patient personality. If none of the items below are particulars for the case please write “all may be used.” | |
| Age range | 30-55 |
| Religious/spiritual background | All may be used |
| Sex (e.g., male, female, intersex, transwoman, transman) | All may be used |
| Sexual Orientation (e.g., heterosexual, lesbian, gay, bisexual, pansexual, queer, asexual) | All may be used |
| Gender expression (e.g., man, woman, gender queer) | All may be used |
| Race/ethnicity: | All may be used |
| Physical description (e.g., BMI, height range) | All may be used |
| Physical limitations | All may be used |
| Patient appearance (e.g., disheveled, hospital gown, business casual, casual) | Hospital gown |
| Moulage + location (e.g., none, bruises, scars, body piercing, tattoos) | None |
| Affect (e.g., pleasant, cooperative) | Initially eager for news, then anxious, then enraged and angry, eventually giving way to sadness or fear if anger is addressed appropriately |
| Family group (e.g., who is family, who they live with) | Has a significant other, relatively new relationship. Recent divorce. All other background may be used. |
| Education | All may be used |
| Level of health literacy | Adequate; had good understanding of surgery that was planned |
| Employment, if any - present and past, noting any current stresses | All may be used |
| Home/homeless - type of dwelling, number of stories, owned or rented | All may be used |
| Financial situation- any current stresses | All may be used |
| Insurance Status (e.g., un/under/insured, public/private, HMO/PPO) | All may be used |
| Habits (i.e., diet, exercise, caffeine, smoking, alcohol, drugs) | All may be used |
| Activities (i.e., hobbies, sports, clubs, friends) | All may be used |
| Typical day - what is the usual daily routine | All may be used |

| CASE INFORMATION | |
| --- | --- |
| Chief Concern: What the patient will say when greeted by the student. The patient’s primary reason for seeking medical care often stated in his/own words. | Patient has just returned from surgery, and is expecting to hear news from the surgeon about how surgery (partial small bowel resection) went. |
| Additional Concerns: Other, if any, concerns the patient has today (i.e., symptoms, requests, expectations, etc.) that will become part of set agenda. |  |
|  | |
| THE PATIENT STORY: The SP will be asked to tell their symptom story and the personal and emotion impact for each of their concerns. You will want to write this is the patient voice. The symptom story should be able to answer this question: “Tell me more about [chief concern/additional concern], starting at the beginning and bringing me up to now.”  The personal context should be able to answer questions concerning the broader personal/psychosocial context of symptoms, especially the patient beliefs/attributions.  The emotional context should be able to ask how are you doing with this, how does this make you feel, how has this affected you emotionally? IMPACT: How has this affected your life? How has this been for your family? | The patient is in a hospital room having just returned from the post-surgery recovery area. The patient underwent a small bowel surgery (“partial small bowel resection”) for Crohn’s disease-related strictures a few hours ago. The patient has recently come out of anesthesia but is fully awake. The surgical resident (the assistant to the attending surgeon) is about to walk in and break the bad news that he/she had to put in an “ileostomy,” rather than the “end-to-end anastomosis” that the patient expected – and was told was the plan. The patient has a good handle on what these terms mean and what was planned - “removing part of my intestine that had a stricture from my Crohn’s disease.”  At the start of the discussion, the patient has not realized that anything went wrong, and simply wants to know how the surgery went, with full expectation that everything went as planned. Although weak from surgery and in pain, upon learning that an ileostomy was placed rather than the planned “end-to-end anastomoses,” the patient becomes livid and responds with anger.  The patient cannot believe how the doctor “lied to me.” He/she “promised me that I wouldn’t need a bag.” The patient’s perspective is that he/she has called the office many times in recent weeks to go over the plan and to ask questions and the patient’s understanding was that it was highly unlikely that “I’d end up with a bag.” The doctor only called back a few times, otherwise the patient always had to speak with the nurse, and the patient wonders if all of the concerns were made clear.  At this point, if the interviewer provides appropriate space for the patient to explain the personal and emotional contexts, rather than taking a defensive stance or diving into a purely medical explanation, the patient can reveal additional relevant details: “I don’t know how I will function. My social life will be ruined. I just began dating again after a messy divorce three years ago. I am afraid this new person won’t want to date me any longer – who would want to date someone with a bag?!”  The patient is afraid of the unknown and also enraged and overwhelmed. The patient should communicate these emotions to the doctor and see if the interviewer responds in a way that makes the patient feel heard, cared for, and understood. If so, the patient should feel the anger give way to another emotion, perhaps sadness or fear, providing opportunity for the interviewer to empathize with these emotions as well. |
| HISTORY OF PRESENT ILLNESS: Although some of the HPI will be given in the patient’s symptom story, the learners will expand the story during the direct question section. Below describe the detailed history, usually about the chief concern, which the student must develop in order to make a useful assessment of the problem: | |
|  | |
| Onset (when; gradual or sudden) | Chronic problem |
| Setting (what was going on or where was patient when symptoms first noticed?) | For years the patient has dealt with abdominal pain, diarrhea (sometimes bloody), and weight loss. This was a huge challenge in previous marriage. Patient has been on multiple drugs for this over the years including methotrexate, infliximab, and others. Patient has had several bowel obstructions and strictures and recently patient and doctors decided that surgery was the only option. |
| Duration (how long) | Chronic (Crohn’s disease for at least 10 years) |
| Time relationships (frequency, constant or intermittent) | Not relevant to case |
| Location | Abdomen |
| Radiation | Not relevant to case |
| Quality | Not relevant to case |
| Amount | Not relevant to case |
| Aggravated by what | Not relevant to case |
| Relieved by what | Not relevant to case |
| Associated with what | Not relevant to case |
| Attitude (what does the patient think is the problem, and how does he/she feel about it) | Not relevant to case |
| Overall course | See above |
| REVIEW OF SYSTEMS: Significant positives and negatives | |
|  | See above |
|  |  |
|  |  |
|  |  |
|  | |
| Past medical history |  |
| Medication allergies (Name and reaction) | All may be used, but not relevant to case |
| Environmental allergies (Name and reaction) | All may be used, but not relevant to case |
| Illnesses | Crohn’s disease for at least 10 years |
| Vaccinations | All may be used, but not relevant to case |
| Surgeries | None prior to current episode |
| Accidents/ injuries/ trauma | All may be used, but not relevant to case |
| Hospitalization | All may be used, but not relevant to case |
|  | |
| Inclusive sexual and reproductive history | |
| Sexual practices  Sexual partners  Protection: Use of safer sex practices  Use of birth control if appropriate  Risk of intimate partner violence | All may be used, but not relevant to case except for concern for impact on intimate relationships (including sexuality) because of ileostomy. |
| Ob/GYN HISTORY | Age of onset of menses --- Not relevant to case  Age of menopause  Number of pregnancies  Number of live births  Number of miscarriages  Number of abortions |
| Medications | Prescription/dose/reason  Over the counter/dose/reason  Herbs/supplements/dose/reason  Other: None presently. Previously prescribed “methotrexate, infliximab, and others” for medical management of Crohn’s disease. |
| Immunizations | - Tetanus --- Not relevant to case - Flu - Hepatitis - Pneumovax - HPV - Other |
| Tobacco products:   - Cigarettes - Cigar - Pipe - Chew - E-cigarettes | - Never --- Not relevant to case - Past- year started/year quit - Current   - Quantity   - # of years |
| Alcohol   - Beer - Wine - Liquor - Other | - Never --- Not relevant to case - Past- year started/year quit - Current   - Quantity   - # of years |
| Drugs   - Weed - Cocaine - Heroin - Meth - Other - IV - Inhalants - Other | - Never --- Not relevant to case - Past- year started/year quit - Current   - Quantity - # of years |
| Diet (describe) | Not relevant to case |
| Exercise (describe) | Not relevant to case |
| List any other important social history or information important to this case | See above |
| Family history |  |
| Mother, Father, Siblings, Grandparents, and other significant findings. | Not relevant to case |
|  |  |
| Physical Exam- List exam maneuvers expected for this case and any abnormal findings that SP will simulate. (tenderness, hyper-hypo reflex, rebound, weakness etc. )  No physical exam as part of this case. | |
| PHYSICAL EXAM FINDINGS |  |
| 1. Written in layman’s terms | N/A |
| 1. General appearance- affect, appearance, position of patient at opening (i.e. sitting, laying down, holding abdomen etc.) | Tired, has just returned to hospital room following surgery. Can be sitting or lying down. Comfortable (pain is controlled). |
| 1. Vital signs | afebrile, P 82, R 12, BP 140/82 |
| 1. Specific findings and affect | See description of emotional reactions above. |
| 1. Response to certain physical movements | Will have minimal movements having just returned from surgery. |
|  |  |
| DIAGNOSIS AND DIFFERENTIAL |  |
| Diagnosis with support from positive and negative history and PE findings | Not relevant to case |
| Differential with support from positive and negative history and PE findings | Not relevant to case |
|  |  |
| MANAGEMENT OR DIAGNOSTIC PLAN | This is a case entirely about communication. The focus should not be on medical details, though if the patient asks about whether ileostomy could be reversed as might occur in the flow of conversation, the interviewer can say that it’s possible but would take at least 3-6 months (or possibly longer) in order to allow any adhesions to soften and decrease risk of injury such as enterotomies. |
|  |  |
| PROFESSIONALISM ISSUES OR CHALLENGES: | The patient is designed to challenge the interviewer, given that the focus of the case is communication with a patient who is demonstrating strong emotions. |
