# Supplementary material for: An Advanced Communication Skills Workshop Using Standardized Patients for Senior Medical Students
Source: MedEdPORTAL. 2021 May 27;17:11163. doi: 10.15766/mep_2374-8265.11163 (PMC8155077; doi:10.15766/mep_2374-8265.11163)
Supplement: Supplementary file 1 — Schedule & Logistics.xlsxStrong Emotion Case Materials.docxGoals of Care Case Materials.docxError Disclosure Case Materials.docxPalliative Care Case Materials.docxStudent Instructions.docxPostsession Survey.docxFaculty Debrief Guide.docx [file mep_2374-8265.11163-s001.zip › C. Goals of Care Case Materials.docx]

**Material for Student Interviewer**

**Setting:** You are a medicine intern. You are talking to the patient’s son/daughter in a family meeting room.

**Opening Scenario (read this carefully before entering the room):**

The patient is an 87-year-old man who was found by his son/daughter at home confused and poorly responsive. Aside from some curative skin cancer excisions and arthritis in the hips and knees, he had been doing well and living independently in a retirement community. He was admitted from the Emergency Department to the Medical Intensive Care Unit 3 days ago.

Currently, the patient is intubated and being treated for pneumonia with bacteremia and persistent rectal bleeding. His kidney function is worsening with increasing creatinine level, and imaging in last 24 hours shows new mesenteric ischemia. The team saw an incidental large colon mass on CT and suspects colon cancer, but given the evolving critical illness, further workup has not been prioritized. The patient is obtunded and unable to participate in the discussion. Your team has witnessed a precipitous decline in the last 12 hours and sees the patient is actively dying with a time frame of hours. Any further intervention will not be helpful. Your team is recommending comfort care at this time.

You are about to have a face to face conversation with his son/daughter for the first time, as they had to travel out of town for work right after the patient was admitted. However the son/daughter is up to speed with the current condition, because they have been receiving updates from you and your team by phone. At the last phone conversation, the son/daughter was told that new imaging showed part of the colon was dying with no intervention possible, and they needed to come in to make important decisions about the goals of care.

**Student Tasks:**

Ensure that the son/daughter has a clear understanding of the father’s clinical situation and discuss goals of care. The patient is actively dying; your priority is to guide the son/daughter toward your team’s plan to focus on comfort as is appropriate in this situation. You have up to 20 minutes with the patient’s son/daughter. You do not need to do a physical exam.

**Self-assessment Communication Behavior Checklist for Student Interviewer**

**Complete the following checklist based on the interview you just performed:**

| 1. I confirmed that son/daughter is health care proxy for father and the correct person to be speaking with. | ( ) Yes | ( ) Partial | ( ) No |
| --- | --- | --- | --- |
| 2. I elicited son’s/daughter’s understanding of father’s current medical condition with open-ended question(s). | ( ) Yes | ( ) Partial | ( ) No |
| 3. I elicited son’s/daughter’s understanding of father’s values and preferences with open-ended question(s). | ( ) Yes | ( ) Partial | ( ) No |
| 4. I described father’s current medical condition with jargon-free language. | ( ) Yes | ( ) Partial | ( ) No |
| 5. I informed son/daughter about prognosis of imminent death with direct, jargon free language. | ( ) Yes | ( ) Partial | ( ) No |
| 6. I discussed resuscitation preferences/DNR orders with non-mechanistic simple language. | ( ) Yes | ( ) Partial | ( ) No |
| 7. I did NOT say, “Do you want us to do everything?” | ( ) Yes | ( ) Partial | ( ) No |
| 8. I responded to son/daughter’s emotion with verbal empathic statement(s). | ( ) Yes | ( ) Partial | ( ) No |
| 9. I responded to son/daughter’s emotion with non-verbal empathic behaviors (e.g. offering tissues, leaning in, light touch, respectful silence). | ( ) Yes | ( ) Partial | ( ) No |
| 10. When asked, I made a recommendation that focus of care be comfort and a Do Not Resuscitate order be enacted. | ( ) Yes | ( ) Partial | ( ) No |
| 11. I informed son/daughter that goals of care will include all efforts to assure as much comfort as possible for father. | ( ) Yes | ( ) Partial | ( ) No |

**Material for Student Observers (also suitable for other assessors – SPs, faculty, etc.)**

**Tasks for Student Observers:**  You will observe a classmate having a conversation with a patient’s family member. Complete the history checklist on the next page as you observe your classmate and be prepared to provide feedback at the end of the 20 minute interview. Your classmate has been given the following instructions.

**Setting:** You are a medicine intern. You are talking to the patient’s son/daughter in a family meeting room.

**Opening Scenario (read this carefully before entering the room):**

The patient is an 87-year-old man who was found by his son/daughter at home confused and poorly responsive. Aside from some curative skin cancer excisions and arthritis in the hips and knees, he had been doing well and living independently in a retirement community. He was admitted from the Emergency Department to the Medical Intensive Care Unit 3 days ago.

Currently, the patient is intubated and being treated for pneumonia with bacteremia and persistent rectal bleeding. His kidney function is worsening with increasing creatinine level, and imaging in last 24 hours shows new mesenteric ischemia. The team saw an incidental large colon mass on CT and suspects colon cancer, but given the evolving critical illness, further workup has not been prioritized. The patient is obtunded and unable to participate in the discussion. Your team has witnessed a precipitous decline in the last 12 hrs and sees the patient is actively dying with a time frame of hours. Any further intervention will not be helpful. Your team is recommending comfort care at this time.

You are about to have a face to face conversation with his son/daughter for the first time, as they had to travel out of town for work right after the patient was admitted. However the son/daughter is up to speed with the current condition, because they have been receiving updates from you and your team by phone. At the last phone conversation, the son/daughter was told that new imaging showed part of the colon was dying with no intervention possible, and they needed to come in to make important decisions about the goals of care.

**Student Tasks:**

Ensure that the son/daughter has a clear understanding of the father’s clinical situation and discuss goals of care. The patient is actively dying; your priority is to guide the son/daughter toward your team’s plan to focus on comfort as is appropriate in this situation. You have up to 20 minutes with the patient’s son/daughter. You do not need to do a physical exam.

**Communication Behavior Checklist for Student Observer**

**(also suitable for other assessors – SPs, faculty, etc.)**

**Complete the following checklist during the interview as you observe:**

| 1. Confirms that son/daughter is health care proxy for father and the correct person to be speaking with. | ( ) Yes | ( ) Partial | ( ) No |
| --- | --- | --- | --- |
| 2. Elicits son’s/daughter’s understanding of father’s current medical condition with open-ended question(s). | ( ) Yes | ( ) Partial | ( ) No |
| 3. Elicits son’s/daughter’s understanding of father’s values and preferences with open-ended question(s). | ( ) Yes | ( ) Partial | ( ) No |
| 4. Describes father’s current medical condition with jargon-free language. | ( ) Yes | ( ) Partial | ( ) No |
| 5. Informs son/daughter about prognosis of imminent death with direct, jargon free language. | ( ) Yes | ( ) Partial | ( ) No |
| 6. Discusses resuscitation preferences/DNR orders with non-mechanistic simple language | ( ) Yes | ( ) Partial | ( ) No |
| 7. Does NOT say, “Do you want us to do everything?” | ( ) Yes | ( ) Partial | ( ) No |
| 8. Responds to son/daughter’s emotion with verbal empathic statement(s). | ( ) Yes | ( ) Partial | ( ) No |
| 9. Responds to son/daughter’s emotion with non-verbal empathic behaviors (e.g. offering tissues, leaning in, light touch, respectful silence). | ( ) Yes | ( ) Partial | ( ) No |
| 10. When asked, makes a recommendation that focus of care be comfort and a Do Not Resuscitate order be enacted. | ( ) Yes | ( ) Partial | ( ) No |
| 11. Informs son/daughter that goals of care will include all efforts to assure as much comfort as possible for father. | ( ) Yes | ( ) Partial | ( ) No |

**Modified Master Interview Rating Scale (MIRS) for Student Observer**

**(also suitable for other assessors – SPs, faculty, etc.)**

The full MIRS can be found in Supplement 1 of Baldwin JD, Cox J, Wu ZH, Kenny A, Angus S. Delivery and Measurement of High-Value Care in Standardized Patient Encounters. Journal of Graduate Medical Education. 2017;9:645-449. [https://doi.org/10.4300/JGME-D-17-00016.1](https://nam12.safelinks.protection.outlook.com/?url=https%3A%2F%2Fdoi.org%2F10.4300%2FJGME-D-17-00016.1&data=04%7C01%7Cjaideep.talwalkar%40yale.edu%7Cb3f1b75239754625b97308d8cecb8002%7Cdd8cbebb21394df8b4114e3e87abeb5c%7C0%7C0%7C637486720727321375%7CUnknown%7CTWFpbGZsb3d8eyJWIjoiMC4wLjAwMDAiLCJQIjoiV2luMzIiLCJBTiI6Ik1haWwiLCJXVCI6Mn0%3D%7C3000&sdata=JvGyuedfM5vMJOVISVQZh1SaegcnYwZzlZCp2cdfZbw%3D&reserved=0)

Items from MIRS used by Student Observers:

1. Opening

12. Questioning Skills – Lack of Jargon

14. Interactive Techniques

15. Verbal Faciliation Skills

16. Non-Verbal Facilitation Skills

17. Empathy and Acknowledging Patient Cues

22. Patient’s Education & Understanding

27. Encouragement of Questions

28. Closure

**Case script for Standardized Patient**

**Standardized Patient Name:** use your regular character’s name
**Actor:**

**Age:** use your regular character’s age, 45-65 years old 
**Episode:** Goals of Care
**Workshop:** Advanced Communcation Skills, Fourth Year Capstone Course
____________________________________________________________________________________

You are the son/daughter of the patient, your 87-year-old father, who is in the hospital. Your father is a widower and has been living independently in a retirement community for many years. You are an only child, and you live near your father and check in on him daily, which you’ve done for the last few years. You assist him with all transportation, finances, and medications, and your father has been able to walk with a walker and be independent in self-care. Your mother committed suicide when you were a teenager, but your father has rarely discussed her with you.

You found your father confused and sleepy when you checked in on him 3 days ago and brought him to the Emergency Department and they admitted him to the Medical Intensive Care Unit because he was very sick. You had to leave town for work the next day but have been speaking with the doctors in the hospital by phone every day, sometimes twice a day. They have explained things to you clearly and you have a good understanding of the medical details before today’s in-person meeting* – he has a pneumonia, a blood infection, and is bleeding from his rectum. He is intubated and on a ventilator to support his breathing along with medicines to support his blood pressure. The rectal bleeding is thought to be from a new, large colon mass identified on CT scan but he has been too sick for a biopsy to be considered yet. More recently, his kidney function has worsened very quickly and significantly. And more importantly, the team told you within the last 12 hours that part of his colon has started to die due to lack of blood flow.** He can’t go to surgery because he is too sick and unstable. Today, the team asked you to come in to see your father and discuss his care, expressing concern about any hope for recovery. You returned to town urgently and have come right to the hospital. You know that he is really sick, the sickest he has ever been – “he’s been a very strong guy until now but this is sounding really serious and scary.”

The doctor may ask you about advance directives, a living will, or durable power of attorney for health care, and should explain these terms to you in basic language. You should have a basic understanding about what these terms mean to move the conversation along.*** As far as you are aware your father didn’t have any advance directives, etc. He always spoke about “living every day to the fullest” but has mentioned a few times that he wouldn’t want to suffer like some other family members when his time to die approaches. You are shocked that all this has happened so quickly and don’t want him to die. Until this happened, your father was still enjoying playing cards, watching TV, and spending time with the family. You love having him in your life. However, as long as the doctors assure you that all has been done to try to help him recover, you would want him to die peacefully if it’s an inevitable path. You would look to the doctors to guide you toward the most compassionate and comfortable path for your father’s care. If not reassured when your concerns are expressed, you would continue to look for reassurance and clarification in the midst of distress and great concern.

* Ideally the doctor will ask you about your understanding of the situation, and if they do, you can explain the background details that you had learned over the phone. This is important because it will allow the focus of the in-person meeting to be about the next-steps rather than the doctor providing a detailed explanation of a medical situation that you already understand.

** This is a very important detail; the student should make it clear to you that this update suggests pending death.

*** Explanation - Advance directives are legal documents that allow you to spell out your decisions about end-of-life care ahead of time. They give you a way to tell your wishes to family, friends, and health care professionals and to avoid confusion later on when you may be unable to speak for yourself.

A living will tells which treatments you want if you are dying or permanently unconscious. You can accept or refuse medical care. You might want to include instructions on:

- The use of dialysis and breathing machines
- If you want to be resuscitated if your breathing or heartbeat stops (“DNR”)
- Tube feeding
- Organ or tissue donation

A durable power of attorney for health care is a document that names your health care proxy. Your proxy is someone you trust to make health decisions for you if you are unable to do so.

***MedEdPORTAL* Standardized Patient Case Development Tool**

Date: January 26, 2021

Primary Case Author: Yale School of Medicine Advanced Communication Skills Workshop group

Secondary Case Author: Not applicable

Standardized Patient Educator: Not applicable

Name of Case: Goals of Care

Name of educational and or assessment activity: Advanced Communication Skills Workshop

Patient Name: Character’s regular name

Chief Concern: Goals of Care discussion

Most likely Diagnosis and Differential with rationale from history and/or physical exam: Not applicable

Challenge question:

You are a medicine intern. You are talking to the patient’s son/daughter in a family meeting room.

The patient is an 87-year-old man who was found by his son/daughter at home confused and poorly responsive. Aside from some curative skin cancer excisions and arthritis in the hips and knees, he had been doing well and living independently in a retirement community. He was admitted from the Emergency Department to the Medical Intensive Care Unit 3 days ago.

Currently, the patient is intubated and being treated for pneumonia with bacteremia and persistent rectal bleeding. His kidney function is worsening with increasing creatinine level, and imaging in last 24 hours shows new mesenteric ischemia. The team saw an incidental large colon mass on CT and suspects colon cancer, but given the evolving critical illness, further workup has not been prioritized. The patient is obtunded and unable to participate in the discussion. Your team has witnessed a precipitous decline in the last 12 hours and sees the patient is actively dying with a time frame of hours. Any further intervention will not be helpful. Your team is recommending comfort care at this time.

You are about to have a face to face conversation with his son/daughter for the first time, as they had to travel out of town for work right after the patient was admitted. However the son/daughter is up to speed with the current condition, because they have been receiving updates from you and your team by phone. At the last phone conversation, the son/daughter was told that new imaging showed part of the colon was dying with no intervention possible, and they needed to come in to make important decisions about the goals of care.

Your task is to ensure that the son/daughter has a clear understanding of the father’s clinical situation and discuss goals of care. The patient is actively dying; your priority is to guide the son/daughter toward your team’s plan to focus on comfort as is appropriate in this situation. You have up to 20 minutes with the patient’s son/daughter. You do not need to do a physical exam.

Domains: Check all that apply

- Professionalism
- Communication and Interpersonal skills
- Medical History
- Physical exam
- Shared Decision Making
- Patient Education
- Clinical Reasoning
- Documentation
- Handoff
- Presentation
- Other:

Type and level of learner: Senior medical student

Case Objectives: please list specific objectives for each of the domains you have checked above:

1. Conduct a goals of care conversation with a family member regarding their critically ill loved one

2. Respond to emotions exhibited by the family member with verbal empathic statements

3. Respond to emotions exhibited by the family member with appropriate body language

| SETTING: outpatient, in patient, ED, home, nursing home, rehab, group etc. | Private family meeting room, intensive care unit |
| --- | --- |
| PATIENT PROFILE: Information about the “patient” that helps select an SP and helps the learner get an understanding of them as a person. SP will know more information about the patient than learner will ever ask but allows SP to portray a fully developed patient personality. If none of the items below are particulars for the case please write “all may be used.” | |
| Age range | 45-65 |
| Religious/spiritual background | All may be used |
| Sex (e.g., male, female, intersex, transwoman, transman) | All may be used |
| Sexual Orientation (e.g., heterosexual, lesbian, gay, bisexual, pansexual, queer, asexual) | All may be used |
| Gender expression (e.g., man, woman, gender queer) | All may be used |
| Race/ethnicity: | All may be used |
| Physical description (e.g., BMI, height range) | All may be used |
| Physical limitations | All may be used |
| Patient appearance (e.g., disheveled, hospital gown, business casual, casual) | Business attire; urgently returned to town from business trip and has come right to the hospital. |
| Moulage + location (e.g., none, bruises, scars, body piercing, tattoos) | None |
| Affect (e.g., pleasant, cooperative) | Initially concerned and eager for an update. Upon hearing about the severity of illness and prognosis, the actor is shocked and distressed. If this emotion is handled with understanding and compassion, it can give way to sadness and then lead to a discussion in which the actor shares the patient’s prior expressed wishes and the doctor’s recommendations for compassionate care for the patient. If not handled appropriately, continue to look for reassurance and clarification in the midst of distress and great concern. |
| Family group (e.g., who is family, who they live with) | Actor is the son/daughter of the patient, the 87-year-old father, who is in the hospital. The father is a widower and has been living independently in a retirement community for many years. The actor is an only child, lives near the father and checks in on him daily, which the actor has done for the last few years. The actor assists him with all transportation, finances, and medications, and the father has been able to walk with a walker and be independent in self-care. The actor’s mother committed suicide when the actor was a teenager, but the father has rarely discussed her with the actor. |
| Education | All may be used |
| Level of health literacy | Excellent; has good lay-person’s understanding of medical details that were explained in past few days through series of phone calls. |
| Employment, if any - present and past, noting any current stresses | Non-medical professional returning to town urgently from business trip. |
| Home/homeless - type of dwelling, number of stories, owned or rented | All may be used |
| Financial situation- any current stresses | All may be used |
| Insurance Status (e.g., un/under/insured, public/private, HMO/PPO) | All may be used |
| Habits (i.e., diet, exercise, caffeine, smoking, alcohol, drugs) | All may be used |
| Activities (i.e., hobbies, sports, clubs, friends) | All may be used |
| Typical day - what is the usual daily routine | All may be used |

| CASE INFORMATION | |
| --- | --- |
| Chief Concern: What the patient will say when greeted by the student. The patient’s primary reason for seeking medical care often stated in his/own words. | The actor is the son/daughter of the patient, the 87-year-old father, who is in the hospital. The son/daughter has returned to town urgently from a business trip when the doctor suggested the son/daughter “come in to see my father and discuss his care.” The doctor had expressed concern about any hope for recovery. The doctor who is meeting the son/daughter in person today is the same one who has been providing phone updates over the past few days, but they have never met in person. |
| Additional Concerns: Other, if any, concerns the patient has today (i.e., symptoms, requests, expectations, etc.) that will become part of set agenda. |  |
|  | |
| THE PATIENT STORY: The SP will be asked to tell their symptom story and the personal and emotion impact for each of their concerns. You will want to write this is the patient voice. The symptom story should be able to answer this question: “Tell me more about [chief concern/additional concern], starting at the beginning and bringing me up to now.”  The personal context should be able to answer questions concerning the broader personal/psychosocial context of symptoms, especially the patient beliefs/attributions.  The emotional context should be able to ask how are you doing with this, how does this make you feel, how has this affected you emotionally? IMPACT: How has this affected your life? How has this been for your family? | Instructions and some relevent background information for the actor is as follows: You found your father confused and sleepy when you checked in on him 3 days ago and brought him to the Emergency Department and they admitted him to the Medical Intensive Care Unit because he was very sick. You had to leave town for work the next day but have been speaking with the doctors in the hospital by phone every day, sometimes twice a day. They have explained things to you clearly and you have a good understanding of the medical details before today’s in-person meeting – he has a pneumonia, a blood infection, and is bleeding from his rectum. He is intubated and on a ventilator to support his breathing along with medicines to support his blood pressure. The rectal bleeding is thought to be from a new, large colon mass identified on CT scan but he has been too sick for a biopsy to be considered yet. More recently, his kidney function has worsened very quickly and significantly. And more importantly, the team told you within the last 12 hours that part of his colon has started to die due to lack of blood flow. Your father can’t go to surgery because he is too sick and unstable. Today, the team asked you to come in to see your father and discuss his care, expressing concern about any hope for recovery. You know that he is really sick, the sickest he has ever been – “he’s been a very strong guy until now but this is sounding really serious and scary.”  Ideally the doctor will ask you about your understanding of the situation, and if they do, you can explain the background details that you had learned over the phone. This is important because it will allow the focus of the in-person meeting to be about the next-steps rather than the doctor providing a detailed explanation of a medical situation that you already understand. The last detail about the colon dying is very important; the student should make it clear to you that this update suggests pending death.  The doctor may ask the son/daughter about advance directives (see below for explanation about what this means). As far as you are aware your father didn’t have any advance directives. He always spoke about “living every day to the fullest” but has mentioned a few times that he wouldn’t want to suffer like some other family members when his time to die approaches. You are shocked that all this has happened so quickly and don’t want him to die. Until this happened, your father was still enjoying playing cards, watching TV, and spending time with the family. You love having him in your life. However, as long as the doctors assure you that all has been done to try to help him recover and your sadness is acknowledged, you are able to clarify you would want him to die peacefully if it’s an inevitable path. You would look to the doctors to guide you toward the most compassionate and comfortable path for your father’s care. If not reassured with an empathic approach when your concerns are expressed, you would continue to look for reassurance and clarification in the midst of distress and great concern.  There are some important terms that the doctor might mention. The doctor should explain what these mean but the actor may find it helpful to have a general sense about these concepts to move the conversation along.  The doctor might mention “advance directives,” which are legal documents that allow you to spell out your decisions about end-of-life care ahead of time. They give you a way to tell your wishes to family, friends, and health care professionals and to avoid confusion later on when you may be unable to speak for yourself.  The doctor might mention a “living will,” which tells which treatments you want if you are dying or permanently unconscious. You can accept or refuse medical care. You might want to include instructions on:   - The use of dialysis and breathing machines - If you want to be resuscitated if your breathing or heartbeat stops (“DNR”) - Tube feeding - Organ or tissue donation   The doctor might mention “durable power of attorney for health care” which is a document that names your health care proxy. Your proxy is someone you trust to make health decisions for you if you are unable to do so. |
| HISTORY OF PRESENT ILLNESS: Although some of the HPI will be given in the patient’s symptom story, the learners will expand the story during the direct question section. Below describe the detailed history, usually about the chief concern, which the student must develop in order to make a useful assessment of the problem: | |
|  | |
| Onset (when; gradual or sudden) | Sudden |
| Setting (what was going on or where was patient when symptoms first noticed?) | You found your father confused and sleepy when you checked in on him 3 days ago and brought him to the Emergency Department and they admitted him to the Medical Intensive Care Unit because he was very sick. |
| Duration (how long) | 3 days |
| Time relationships (frequency, constant or intermittent) | Not relevant to case |
| Location | Not relevant to case |
| Radiation | Not relevant to case |
| Quality | Not relevant to case |
| Amount | Not relevant to case |
| Aggravated by what | Not relevant to case |
| Relieved by what | Not relevant to case |
| Associated with what | Not relevant to case |
| Attitude (what does the patient think is the problem, and how does he/she feel about it) | Not relevant to case |
| Overall course | See above |
| REVIEW OF SYSTEMS: Significant positives and negatives | |
|  | Not relevant to case |
|  |  |
|  |  |
|  |  |
|  | |
| Past medical history |  |
| Medication allergies (Name and reaction) | All may be used, but not relevant to case |
| Environmental allergies (Name and reaction) | All may be used, but not relevant to case |
| Illnesses | The important point is that the patient had been generally healthy before the acute illness. He had curative skin cancer excisions and arthritis in the hips and knees, and is on some chronic medications, but the specific details are not relevant to case. |
| Vaccinations | All may be used, but not relevant to case |
| Surgeries | All may be used, but not relevant to case |
| Accidents/ injuries/ trauma | All may be used, but not relevant to case |
| Hospitalization | All may be used, but not relevant to case |
|  | |
| Inclusive sexual and reproductive history | |
| Sexual practices  Sexual partners  Protection: Use of safer sex practices  Use of birth control if appropriate  Risk of intimate partner violence | All may be used, but not relevant to case |
| Ob/GYN HISTORY | Age of onset of menses --- Not relevant to case  Age of menopause  Number of pregnancies  Number of live births  Number of miscarriages  Number of abortions |
| Medications | Prescription/dose/reason --- on some chronic medications for chronic conditions but details are not relevant to case  Over the counter/dose/reason  Herbs/supplements/dose/reason  Other: |
| Immunizations | - Tetanus --- Not relevant to case - Flu - Hepatitis - Pneumovax - HPV - Other |
| Tobacco products:   - Cigarettes - Cigar - Pipe - Chew - E-cigarettes | - Never --- Not relevant to case - Past- year started/year quit - Current   - Quantity   - # of years |
| Alcohol   - Beer - Wine - Liquor - Other | - Never --- Not relevant to case - Past- year started/year quit - Current   - Quantity   - # of years |
| Drugs   - Weed - Cocaine - Heroin - Meth - Other - IV - Inhalants - Other | - Never --- Not relevant to case - Past- year started/year quit - Current   - Quantity - # of years |
| Diet (describe) | Not relevant to case |
| Exercise (describe) | Not relevant to case |
| List any other important social history or information important to this case | See above |
| Family history |  |
| Mother, Father, Siblings, Grandparents, and other significant findings. | Actor’s mother (the patient’s wife) committed suicide when actor was a teenager, but the father has rarely discussed her. |
|  |  |
| Physical Exam- List exam maneuvers expected for this case and any abnormal findings that SP will simulate. (tenderness, hyper-hypo reflex, rebound, weakness etc. )  No physical exam as part of this case. | |
| PHYSICAL EXAM FINDINGS |  |
| 1. Written in layman’s terms | N/A |
| 1. General appearance- affect, appearance, position of patient at opening (i.e. sitting, laying down, holding abdomen etc.) | Appears well, the actor in this case is not seeking healthcare as outlined above |
| 1. Vital signs | N/A |
| 1. Specific findings and affect | See description of emotional reactions above. |
| 1. Response to certain physical movements | Not releveant to case |
|  |  |
| DIAGNOSIS AND DIFFERENTIAL |  |
| Diagnosis with support from positive and negative history and PE findings | Not relevant to case |
| Differential with support from positive and negative history and PE findings | Not relevant to case |
|  |  |
| MANAGEMENT OR DIAGNOSTIC PLAN | This is a case entirely about communication. The focus should not be on medical details, since the son/daughter should already be versed on the basic situation. Instead, ideally the doctor will focus on recognizing and empathizing with emotion, and exploring values and preferences related to goals of care. |
|  |  |
| PROFESSIONALISM ISSUES OR CHALLENGES: | Rather than focus on medical details, the interviewer must recognize and empathize with emotion. If done adequately, this will allow a transition to explore values and preferences related to goals of care. |
