# Supplementary material for: An Advanced Communication Skills Workshop Using Standardized Patients for Senior Medical Students
Source: MedEdPORTAL. 2021 May 27;17:11163. doi: 10.15766/mep_2374-8265.11163 (PMC8155077; doi:10.15766/mep_2374-8265.11163)
Supplement: Supplementary file 1 — Schedule & Logistics.xlsxStrong Emotion Case Materials.docxGoals of Care Case Materials.docxError Disclosure Case Materials.docxPalliative Care Case Materials.docxStudent Instructions.docxPostsession Survey.docxFaculty Debrief Guide.docx [file mep_2374-8265.11163-s001.zip › D. Error Disclosure Case Materials.docx]

**Material for Student Interviewer**

**Setting:** You are a radiology-oncology prelimary resident rotating on the inpatient medicine service.

**Opening Scenario (read this carefully before entering the room):**

The patient is a __-year-old man/woman with hypertension, epigastric pain, and mild shortness of breath. He/she has been otherwise healthy.

He/she came to the ED yesterday afternoon, where workup showed mild leukocytosis and normal chemistries. The original ED plan was to discharge him/her home with a working diagnosis of “dyspepsia” and outpatient follow-up, but after 3 hours he/she complained of increased pain. The ED doctors changed the plan to bring the patient into the hospital on “observation status.”

You examined him/her at 9 pm last night, shortly after he/she arrived on the floor. He/she was in modest discomfort.

Physical Exam at that time: afebrile, R 24, HR 92, BP 150/90

Abdomen: modestly tender in epigastrium, no rebound tenderness. Bowel sounds – occasional tinkles

Meds: hydrochlorothiazide 25 mg daily, atorvastatin 20 mg daily

You ordered an abdominal X-ray and continued your work until about 4 am when you lay down for some sleep before rounds. You were awakened at 6:30 am by a nurse – your patient was much worse. His/her temperature was 101 and he/she now had marked epigastric tenderness. You realized that you never heard about the X-ray you had ordered. When you looked at the film, you were alarmed to see evidence of air under the diaphragm. The patient was taken to the OR at 10 am, where a perforated duodenal ulcer was found. The time between the X-ray and surgery was 12 hours.

It is now about 10 hours after the surgery and you have returned for another overnight shift. The patient is back in a hospital room, now on the surgery service. You are no longer directly involved in his/her care.

**Student Tasks:**

Explain to the patient why there was a delay in diagnosing the perforated duodenal ulcer and getting to surgery. You have up to 20 minutes with the patient to describe what happened, hear the patient’s response and concerns, apologize for the error causing the delay, and plan for follow-up. You do not need to do a physical exam.

**Self-assessment Communication Behavior Checklist for Student Interviewer**

**Complete the following checklist based on the interview you just performed:**

| 1. I asked about patient’s understanding of what happened. | ( ) Yes | ( ) Partial | ( ) No |
| --- | --- | --- | --- |
| 2. I described in clear language what happened. | ( ) Yes | ( ) Partial | ( ) No |
| 3. I described why the error occurred. | ( ) Yes | ( ) Partial | ( ) No |
| 4. I clearly stated what is known and what is not known (at this time) | ( ) Yes | ( ) Partial | ( ) No |
| 5. I stated a clear apology. | ( ) Yes | ( ) Partial | ( ) No |
| 6. I took responsibility. | ( ) Yes | ( ) Partial | ( ) No |
| 7. I discussed steps in future to avoid a similar error. | ( ) Yes | ( ) Partial | ( ) No |
| 8. I discussed follow-up plans, including invitation for further discussions. | ( ) Yes | ( ) Partial | ( ) No |
| 9. I inquired if patient would like me to speak with anyone else about the situation (e.g., family). | ( ) Yes | ( ) Partial | ( ) No |

**Material for Student Observers (also suitable for other assessors – SPs, faculty, etc.)**

**Tasks for Student Observers:**  You will observe a classmate having a conversation with a patient. Complete the history checklist on the next page as you observe your classmate and be prepared to provide feedback at the end of the 20 minute interview. Your classmate has been given the following instructions.

**Setting:** You are a radiology-oncology prelimary resident rotating on the inpatient medicine service.

**Opening Scenario (read this carefully before entering the room):**

The patient is a __-year-old man/woman with hypertension, epigastric pain, and mild shortness of breath. He/she has been otherwise healthy.

He/she came to the ED yesterday afternoon, where workup showed mild leukocytosis and normal chemistries. The original ED plan was to discharge him/her home with a working diagnosis of “dyspepsia” and outpatient follow-up, but after 3 hours he/she complained of increased pain. The ED doctors changed the plan to bring the patient into the hospital on “observation status.”

You examined him/her at 9 pm last night, shortly after he/she arrived on the floor. He/she was in modest discomfort.

Physical Exam at that time: afebrile, R 24, HR 92, BP 150/90

Abdomen: modestly tender in epigastrium, no rebound tenderness. Bowel sounds – occasional tinkles

Meds: hydrochlorothiazide 25 mg daily, atorvastatin 20 mg daily

You ordered an abdominal X-ray and continued your work until about 4 am when you lay down for some sleep before rounds. You were awakened at 6:30 am by a nurse – your patient was much worse. His/her temperature was 101 and he/she now had marked epigastric tenderness. You realized that you never heard about the X-ray you had ordered. When you looked at the film, you were alarmed to see evidence of air under the diaphragm. The patient was taken to the OR at 10 am, where a perforated duodenal ulcer was found. The time between the X-ray and surgery was 12 hours.

It is now about 10 hours after the surgery and you have returned for another overnight shift. The patient is back in a hospital room, now on the surgery service. You are no longer directly involved in his/her care.

**Student Tasks:**

Explain to the patient why there was a delay in diagnosing the perforated duodenal ulcer and getting to surgery. You have up to 20 minutes with the patient to describe what happened, hear the patient’s response and concerns, apologize for the error causing the delay, and plan for follow-up. You do not need to do a physical exam.

**Communication Behavior Checklist for Student Observer**

**(also suitable for other assessors – SPs, faculty, etc.)**

**Complete the following checklist during the interview as you observe:**

| 1. Asks about patient’s understanding of what happened. | ( ) Yes | ( ) Partial | ( ) No |
| --- | --- | --- | --- |
| 2. Describes in clear language what happened. | ( ) Yes | ( ) Partial | ( ) No |
| 3. Describes why the error occurred. | ( ) Yes | ( ) Partial | ( ) No |
| 4. Clearly states what is known and what is not known (at this time) | ( ) Yes | ( ) Partial | ( ) No |
| 5. States a clear apology. | ( ) Yes | ( ) Partial | ( ) No |
| 6. Takes responsibility. | ( ) Yes | ( ) Partial | ( ) No |
| 7. Discusses steps in future to avoid a similar error. | ( ) Yes | ( ) Partial | ( ) No |
| 8. Discusses follow-up plans, including invitation for further discussions. | ( ) Yes | ( ) Partial | ( ) No |
| 9. Inquires if patient would like doctor to speak with anyone else about the situation (e.g., family). | ( ) Yes | ( ) Partial | ( ) No |

**Modified Master Interview Rating Scale (MIRS) for Student Observer**

**(also suitable for other assessors – SPs, faculty, etc.)**

The full MIRS can be found in Supplement 1 of Baldwin JD, Cox J, Wu ZH, Kenny A, Angus S. Delivery and Measurement of High-Value Care in Standardized Patient Encounters. Journal of Graduate Medical Education. 2017;9:645-449. [https://doi.org/10.4300/JGME-D-17-00016.1](https://nam12.safelinks.protection.outlook.com/?url=https%3A%2F%2Fdoi.org%2F10.4300%2FJGME-D-17-00016.1&data=04%7C01%7Cjaideep.talwalkar%40yale.edu%7Cb3f1b75239754625b97308d8cecb8002%7Cdd8cbebb21394df8b4114e3e87abeb5c%7C0%7C0%7C637486720727321375%7CUnknown%7CTWFpbGZsb3d8eyJWIjoiMC4wLjAwMDAiLCJQIjoiV2luMzIiLCJBTiI6Ik1haWwiLCJXVCI6Mn0%3D%7C3000&sdata=JvGyuedfM5vMJOVISVQZh1SaegcnYwZzlZCp2cdfZbw%3D&reserved=0)

Items from MIRS used by Student Observers:

1. Opening

12. Questioning Skills – Lack of Jargon

14. Interactive Techniques

15. Verbal Faciliation Skills

16. Non-Verbal Facilitation Skills

17. Empathy and Acknowledging Patient Cues

22. Patient’s Education & Understanding

27. Encouragement of Questions

28. Closure

**Case script for Standardized Patient**

**Standardized Patient Name:** use your regular character’s name
**Actor:**

**Age:** 35-75
**Episode:** Disclosure of Error
**Workshop:** Advanced Communcation Skills, Fourth Year Capstone Course
____________________________________________________________________________________

You are a patient in a hospital room.

Here is the timeline of your story:

- 2 days ago you began to have some discomfort, a little pain, in your belly.
- Yesterday at 3 pm you went to the hospital emergency room and waited 3 hours to be seen.
- While being examined you had blood tests done and they told you that if the blood tests were normal, you could go home with an appointment to see a doctor in the clinic.
- While waiting for the results, the pain got a little worse. A doctor examined you again, said he thought you were OK, but agreed to “watch you for a while” so they admitted you to the hospital where you met more doctors.
- At about 10 pm they sent you for an Xray of your belly.
- You tried to sleep, but the pain in your belly kept you awake.
- About 6 am, the pain got much worse. You called the nurse.
- A doctor came back to see you at 6:30 am and said that the Xray showed a problem in the belly that might require surgery.
- At 10am you were taken to the operating room.

Now it is evening, and you are recovering from surgery. The surgeon told you that you had an ulcer, which had caused a small hole in your bowel, and that this was repaired with surgery. You need to be in the hospital for a few more days, get IVs and antibiotics, and not eat for a few days. The good news is that the surgeons expect you will recover completely.

You are relieved that you will be OK, but thinking back, you wonder about the sudden concern of the doctors this morning and the emergency surgery. You had an Xray at 10 pm and got worse for hours after it was done, but the doctor only told you the Xray was bad in the morning. Why did it take more than 8 hours to find out that you needed emergency surgery?

Background: You do not see doctors regularly, and you felt perfectly healthy until this belly pain started. You did go to the medical clinic at the hospital last year, and received some medicines for your blood pressure and cholesterol. You did not come back to see the doctor, but your medicine is running out and they told you that you could not get more refills without another visit to the clinic. All these doctors, nurses, and tests at the hospital are a little scary. Fill in other details that come up as you see fit.

A doctor is coming to your room to talk to you. This is one of the many doctors you have seen, and is the one who told you about the Xray results early this morning. Your character does not yet know this, but the doctor’s task is to disclose to you that an error was made (this doctor did not check the Xray results which is what accounted for the delay). You should respond initially with confusion (“I don’t understand… how could something like this happen?”), slight anger (“I was in agony… how could you have left me that way… I could have died”), and ambivalence (“I thought I was getting good care… I don’t know how to feel about this… this is why I don’t like going to doctors.”). Ideally the doctor will address your emotions, apologize, and take responsibility, in which case your initial emotions will give way to gratitude that everything is ok and appreciation for the doctor’s honesty. If the doctor is evasive, defensive, makes excuses, or takes any approach that seems inappropriate to your character, you can prolong your confusion and anger, and retreat to a withdrawn affect rather than having an outburst of emotion.

***MedEdPORTAL* Standardized Patient Case Development Tool**

Date: January 26, 2021

Primary Case Author: Yale School of Medicine Advanced Communication Skills Workshop group

Secondary Case Author: Not applicable

Standardized Patient Educator: Not applicable

Name of Case: Disclosure of Error

Name of educational and or assessment activity: Advanced Communication Skills Workshop

Patient Name: Character’s regular name

Chief Concern: Post-op from perforated ulcer repair

Most likely Diagnosis and Differential with rationale from history and/or physical exam: Not applicable

Challenge question:

You are a radiology-oncology prelimary resident rotating on the inpatient medicine service.

The patient is a (insert actor’s age)-year-old man/woman with hypertension, epigastric pain, and mild shortness of breath. He/she has been otherwise healthy.

He/she came to the ED yesterday afternoon, where workup showed mild leukocytosis and normal chemistries. The original ED plan was to discharge him/her home with a working diagnosis of “dyspepsia” and outpatient follow-up, but after 3 hours he/she complained of increased pain. The ED doctors changed the plan to bring the patient into the hospital on “observation status.”

You examined him/her at 9 pm last night, shortly after he/she arrived on the floor. He/she was in modest discomfort.

Physical Exam at that time: afebrile, R 24, HR 92, BP 150/90

Abdomen: modestly tender in epigastrium, no rebound tenderness. Bowel sounds – occasional tinkles

Meds: hydrochlorothiazide 25 mg daily, atorvastatin 20 mg daily

You ordered an abdominal X-ray and continued your work until about 4 am when you lay down for some sleep before rounds. You were awakened at 6:30 am by a nurse – your patient was much worse. His/her temperature was 101 and he/she now had marked epigastric tenderness. You realized that you never heard about the X-ray you had ordered. When you looked at the film, you were alarmed to see evidence of air under the diaphragm. The patient was taken to the OR at 10 am, where a perforated duodenal ulcer was found. The time between the X-ray and surgery was 12 hours.

It is now about 10 hours after the surgery and you have returned for another overnight shift. The patient is back in a hospital room, now on the surgery service. You are no longer directly involved in his/her care.

Your task is to explain to the patient why there was a delay in diagnosing the perforated duodenal ulcer and getting to surgery. You have up to 20 minutes with the patient to describe what happened, hear the patient’s response and concerns, apologize for the error causing the delay, and plan for follow-up. You do not need to do a physical exam.

Domains: Check all that apply

- Professionalism
- Communication and Interpersonal skills
- Medical History
- Physical exam
- Shared Decision Making
- Patient Education
- Clinical Reasoning
- Documentation
- Handoff
- Presentation
- Other:

Type and level of learner: Senior medical student

Case Objectives: please list specific objectives for each of the domains you have checked above:

1. Disclose a medical error to a patient

2. Respond to emotions exhibited by a patient with verbal empathic statements

3. Respond to emotions exhibited by a patient with appropriate body language

| SETTING: outpatient, in patient, ED, home, nursing home, rehab, group etc. | Hospital room, surgical unit |
| --- | --- |
| PATIENT PROFILE: Information about the “patient” that helps select an SP and helps the learner get an understanding of them as a person. SP will know more information about the patient than learner will ever ask but allows SP to portray a fully developed patient personality. If none of the items below are particulars for the case please write “all may be used.” | |
| Age range | 35-75 |
| Religious/spiritual background | All may be used |
| Sex (e.g., male, female, intersex, transwoman, transman) | All may be used |
| Sexual Orientation (e.g., heterosexual, lesbian, gay, bisexual, pansexual, queer, asexual) | All may be used |
| Gender expression (e.g., man, woman, gender queer) | All may be used |
| Race/ethnicity: | All may be used |
| Physical description (e.g., BMI, height range) | All may be used |
| Physical limitations | All may be used |
| Patient appearance (e.g., disheveled, hospital gown, business casual, casual) | Hospital gown |
| Moulage + location (e.g., none, bruises, scars, body piercing, tattoos) | None |
| Affect (e.g., pleasant, cooperative) | Resting calmly initially. Upon learning of the error, respond with confusion (“I don’t understand… how could something like this happen?”), slight anger (“I was in agony… how could you have left me that way… I could have died”), and ambivalence (“I thought I was getting good care… I don’t know how to feel about this… this is why I don’t like going to doctors.”). Ideally the doctor will address the emotions, apologize, and take responsibility, in which case the initial emotions will give way to gratitude that everything is ok and appreciation for the doctor’s honesty. If the doctor is evasive, defensive, makes excuses, or takes any approach that seems inappropriate to the patient, prolong the confusion and anger, and retreat to a withdrawn affect rather than having an outburst of emotion. |
| Family group (e.g., who is family, who they live with) | All may be used |
| Education | All may be used |
| Level of health literacy | Adequate, though events since arrival to hospital are a blur and have left the patient confused about the timeline of decision making. |
| Employment, if any - present and past, noting any current stresses | All may be used |
| Home/homeless - type of dwelling, number of stories, owned or rented | All may be used |
| Financial situation- any current stresses | All may be used |
| Insurance Status (e.g., un/under/insured, public/private, HMO/PPO) | All may be used |
| Habits (i.e., diet, exercise, caffeine, smoking, alcohol, drugs) | All may be used |
| Activities (i.e., hobbies, sports, clubs, friends) | All may be used |
| Typical day - what is the usual daily routine | All may be used |

| CASE INFORMATION | |
| --- | --- |
| Chief Concern: What the patient will say when greeted by the student. The patient’s primary reason for seeking medical care often stated in his/own words. | Patient is resting in room following emergency surgery for perforated ulcer, has seen many doctors, and has no specific concern, though has been wondering about the long delay between the Xray and being told that emergency surgery was needed. |
| Additional Concerns: Other, if any, concerns the patient has today (i.e., symptoms, requests, expectations, etc.) that will become part of set agenda. |  |
|  | |
| THE PATIENT STORY: The SP will be asked to tell their symptom story and the personal and emotion impact for each of their concerns. You will want to write this is the patient voice. The symptom story should be able to answer this question: “Tell me more about [chief concern/additional concern], starting at the beginning and bringing me up to now.”  The personal context should be able to answer questions concerning the broader personal/psychosocial context of symptoms, especially the patient beliefs/attributions.  The emotional context should be able to ask how are you doing with this, how does this make you feel, how has this affected you emotionally? IMPACT: How has this affected your life? How has this been for your family? | The timeline of events from the patient’s perspective is as follows:   - 2 days ago you began to have some discomfort, a little pain, in your belly. - Yesterday at 3 pm you went to the hospital emergency room and waited 3 hours to be seen. - While being examined you had blood tests done and they told you that if the blood tests were normal, you could go home with an appointment to see a doctor in the clinic. - While waiting for the results, the pain got a little worse. A doctor examined you again, said he thought you were OK, but agreed to “watch you for a while” so they admitted you to the hospital where you met more doctors. - At about 10 pm they sent you for an Xray of your belly. - You tried to sleep, but the pain in your belly kept you awake. - About 6 am, the pain got much worse. You called the nurse. - A doctor came back to see you at 6:30 am and said that the Xray showed a problem in the belly that might require surgery. - At 10am you were taken to the operating room.   Now it is evening, and you are recovering from surgery. The surgeon told you that you had an ulcer, which had caused a small hole in your bowel, and that this was repaired with surgery. You need to be in the hospital for a few more days, get IVs and antibiotics, and not eat for a few days. The good news is that the surgeons expect you will recover completely.  You are relieved that you will be OK, but thinking back, you wonder about the sudden concern of the doctors this morning and the emergency surgery. You had an Xray at 10 pm and got worse for hours after it was done, but the doctor only told you the Xray was bad in the morning. Why did it take more than 8 hours to find out that you needed emergency surgery?  By way of background, you do not see doctors regularly, and you felt perfectly healthy until this belly pain started. You did go to the medical clinic at the hospital last year, and received some medicines for your blood pressure and cholesterol. You did not come back to see the doctor, but your medicine is running out and they told you that you could not get more refills without another visit to the clinic. All these doctors, nurses, and tests at the hospital are a little scary. Fill in other details that come up as you see fit.  The timeline of events from the doctor’s perspective is as follows:  This patient came to the ED yesterday afternoon, where workup showed mild leukocytosis and normal chemistries. The original ED plan was to discharge him/her home with a working diagnosis of “dyspepsia” and outpatient follow-up, but after 3 hours he/she complained of increased pain. The ED doctors changed the plan to bring the patient into the hospital on “observation status.”  You examined him/her at 9 pm last night, shortly after he/she arrived on the floor. He/she was in modest discomfort.  Physical Exam at that time: afebrile, R 24, HR 92, BP 150/90  Abdomen: modestly tender in epigastrium, no rebound tenderness. Bowel sounds – occasional tinkles  Meds: hydrochlorothiazide 25 mg daily, atorvastatin 20 mg daily  You ordered an abdominal X-ray and continued your work until about 4 am when you lay down for some sleep before rounds. You were awakened at 6:30 am by a nurse – your patient was much worse. His/her temperature was 101 and he/she now had marked epigastric tenderness. You realized that you never heard about the X-ray you had ordered. When you looked at the film, you were alarmed to see evidence of air under the diaphragm. The patient was taken to the OR at 10 am, where a perforated duodenal ulcer was found. The time between the X-ray and surgery was 12 hours.  It is now about 10 hours after the surgery and you have returned for another overnight shift. The patient is back in a hospital room, now on the surgery service. You are no longer directly involved in his/her care. |
| HISTORY OF PRESENT ILLNESS: Although some of the HPI will be given in the patient’s symptom story, the learners will expand the story during the direct question section. Below describe the detailed history, usually about the chief concern, which the student must develop in order to make a useful assessment of the problem: | |
|  | |
| Onset (when; gradual or sudden) | Onset two days ago, gradually worsening |
| Setting (what was going on or where was patient when symptoms first noticed?) | Not relevant to case |
| Duration (how long) | 2 days |
| Time relationships (frequency, constant or intermittent) | Not relevant to case |
| Location | Abdomen |
| Radiation | Not relevant to case |
| Quality | Not relevant to case |
| Amount | Not relevant to case |
| Aggravated by what | Not relevant to case |
| Relieved by what | Not relevant to case |
| Associated with what | Not relevant to case |
| Attitude (what does the patient think is the problem, and how does he/she feel about it) | Not relevant to case |
| Overall course | See above |
| REVIEW OF SYSTEMS: Significant positives and negatives | |
|  | See above |
|  |  |
|  |  |
|  |  |
|  | |
| Past medical history |  |
| Medication allergies (Name and reaction) | All may be used, but not relevant to case |
| Environmental allergies (Name and reaction) | All may be used, but not relevant to case |
| Illnesses | Hypertension, high cholesterol |
| Vaccinations | All may be used, but not relevant to case |
| Surgeries | None prior to current episode |
| Accidents/ injuries/ trauma | All may be used, but not relevant to case |
| Hospitalization | All may be used, but not relevant to case |
|  | |
| Inclusive sexual and reproductive history | |
| Sexual practices  Sexual partners  Protection: Use of safer sex practices  Use of birth control if appropriate  Risk of intimate partner violence | All may be used, but not relevant to case. |
| Ob/GYN HISTORY | Age of onset of menses --- Not relevant to case  Age of menopause  Number of pregnancies  Number of live births  Number of miscarriages  Number of abortions |
| Medications | Prescription/dose/reason  Medicines at home: hydrochlorothiazide 25 mg daily for hypertension, atorvastatin 20 mg daily for hyperlipidemia. The patient is not sure of what medicines are being administered in the hospital.  Over the counter/dose/reason  Herbs/supplements/dose/reason  Other: |
| Immunizations | - Tetanus --- Not relevant to case - Flu - Hepatitis - Pneumovax - HPV - Other |
| Tobacco products:   - Cigarettes - Cigar - Pipe - Chew - E-cigarettes | - Never --- Not relevant to case - Past- year started/year quit - Current   - Quantity   - # of years |
| Alcohol   - Beer - Wine - Liquor - Other | - Never --- Not relevant to case - Past- year started/year quit - Current   - Quantity   - # of years |
| Drugs   - Weed - Cocaine - Heroin - Meth - Other - IV - Inhalants - Other | - Never --- Not relevant to case - Past- year started/year quit - Current   - Quantity - # of years |
| Diet (describe) | Not relevant to case |
| Exercise (describe) | Not relevant to case |
| List any other important social history or information important to this case | Not relevant to case |
| Family history |  |
| Mother, Father, Siblings, Grandparents, and other significant findings. | Not relevant to case |
|  |  |
| Physical Exam- List exam maneuvers expected for this case and any abnormal findings that SP will simulate. (tenderness, hyper-hypo reflex, rebound, weakness etc. )  No physical exam as part of this case. Some background physical exam information provided to interviewing student as part of historical case background (see above). | |
| PHYSICAL EXAM FINDINGS |  |
| 1. Written in layman’s terms | N/A |
| 1. General appearance- affect, appearance, position of patient at opening (i.e. sitting, laying down, holding abdomen etc.) | Tired, resting in hospital room following surgery. Can be sitting or laying down. Comfortable (pain is controlled). |
| 1. Vital signs | Not provided, presumed stable. |
| 1. Specific findings and affect | See description of emotional reactions above. |
| 1. Response to certain physical movements | Will have minimal movements having recently returned from surgery. |
|  |  |
| DIAGNOSIS AND DIFFERENTIAL |  |
| Diagnosis with support from positive and negative history and PE findings | Not relevant to case |
| Differential with support from positive and negative history and PE findings | Not relevant to case |
|  |  |
| MANAGEMENT OR DIAGNOSTIC PLAN | This is a case entirely about communication. The focus should be on disclosure of medical error rather than on details of the medical history. The relevant medical details are provided to the interviewer before entering the room. Ideally the doctor will address the patient’s emotions, apologize, and take responsibility for the error. |
|  |  |
| PROFESSIONALISM ISSUES OR CHALLENGES: | The interviewer must exhibit best practices as relate to disclosure of error for success in this station. Ideally the doctor will address the patient’s emotions, apologize, and take responsibility. If done correctly, the patient’s initial emotions (confusion, anger, ambivalence) will give way to gratitude that everything is ok and appreciation for the doctor’s honesty. If the doctor is evasive, defensive, makes excuses, or takes any approach that seems inappropriate to the character, the confusion and anger are prolonged. |
