# Supplementary material for: An Advanced Communication Skills Workshop Using Standardized Patients for Senior Medical Students
Source: MedEdPORTAL. 2021 May 27;17:11163. doi: 10.15766/mep_2374-8265.11163 (PMC8155077; doi:10.15766/mep_2374-8265.11163)
Supplement: Supplementary file 1 — Schedule & Logistics.xlsxStrong Emotion Case Materials.docxGoals of Care Case Materials.docxError Disclosure Case Materials.docxPalliative Care Case Materials.docxStudent Instructions.docxPostsession Survey.docxFaculty Debrief Guide.docx [file mep_2374-8265.11163-s001.zip › E. Palliative Care Case Materials.docx]

**Material for Student Interviewer**

**Setting:** You are a medicine intern in the primary care clinic. This patient has been coming to the clinic for 5 years for treatment of his/her hypertension, but you have not personally met the patient before.

**Opening Scenario (read this carefully before entering the room):**

The patient is a ___-year-old man/woman who was diagnosed with pancreatic carcinoma 7 months ago. He/she has received 2 different chemotherapy drugs over 6 months and neither worked. He/she received the most recent chemotherapy 3 weeks ago, but the cancer has continued to spread. His/her oncologist has said there are no other treatment options for the cancer. You have read a report from the oncologist that states he/she knows the full extent of his/her disease and that no further cancer specific treatment is available.

Vital Signs:  T: 37.1C, P: 76, R: 16, BP 95/60, Pulse Ox: 99% on Room Air
Weight/Height/BMI: (insert actors’s weight/height/BMI)
PMH:      hypertension
Meds:    lisinopril 40 mg daily, oxycodone 5 mg po q4 hours prn pain, ondansetron 4 mg po q4 hours prn

nausea

**Student Tasks:**

Take a palliative care history from a patient who has been told there are no more cancer treatments available for him/her. You have up to 20 minutes with the patient to gather the necessary information to form a palliative care plan for the patient. You do not need to do a physical exam.

**Communication Behavior Checklist for Student Interviewer**

**Complete the following checklist based on the interview you just performed:**

| 1. I opened up dialogue to explore goals of care. | ( ) Yes | ( ) Partial | ( ) No |
| --- | --- | --- | --- |
| 2. I elicited patient’s understanding of disease and prognosis. | ( ) Yes | ( ) Partial | ( ) No |
| 3. I inquired about pain and pain management. | ( ) Yes | ( ) Partial | ( ) No |
| 4. I inquired about nausea or other common physical symptoms in patients with palliative care needs (e.g. weight loss, fatigue). | ( ) Yes | ( ) Partial | ( ) No |
| 5. I inquired in open-ended fashion about physical feelings (and/or noting low blood pressure). | ( ) Yes | ( ) Partial | ( ) No |
| 6. I explored presence of depression/anxiety. | ( ) Yes | ( ) Partial | ( ) No |
| 7. I explored presence of existential concerns/distress. | ( ) Yes | ( ) Partial | ( ) No |
| 8. I asked whether patient is using any complementary treatment. | ( ) Yes | ( ) Partial | ( ) No |
| 9. I inquired about living situation and social supports. | ( ) Yes | ( ) Partial | ( ) No |
| 10. I inquired about family or financial concerns. | ( ) Yes | ( ) Partial | ( ) No |
| 11. I inquired about health care proxy. | ( ) Yes | ( ) Partial | ( ) No |
| 12. I inquired about whether patient has a living will or end-of-life preferences. | ( ) Yes | ( ) Partial | ( ) No |
| 13. I inquired about spirituality or religion. | ( ) Yes | ( ) Partial | ( ) No |
| 14. I explored whether patient knows about hospice. | ( ) Yes | ( ) Partial | ( ) No |

**Material for Student Observers (also suitable for other assessors – SPs, faculty, etc.)**

**Tasks for Student Observers:**  You will observe a classmate having a conversation with a patient. Complete the history checklist on the next page as you observe your classmate and be prepared to provide feedback at the end of the 20 minute interview. Your classmate has been given the following instructions.

**Setting:** You are a medicine intern in the primary care clinic. This patient has been coming to the clinic for 5 years for treatment of his/her hypertension, but you have not personally met the patient before.

**Opening Scenario (read this carefully before entering the room):**

The patient is a ___-year-old man/woman who was diagnosed with pancreatic carcinoma 7 months ago. He/she has received 2 different chemotherapy drugs over 6 months and neither worked. He/she received the most recent chemotherapy 3 weeks ago, but the cancer has continued to spread. His/her oncologist has said there are no other treatment options for the cancer. You have read a report from the oncologist that states he/she knows the full extent of his/her disease and that no further cancer specific treatment is available.

Vital Signs:  T: 37.1C, P: 76, R: 16, BP 95/60, Pulse Ox: 99% on Room Air
Weight/Height/BMI: (insert actors’s weight/height/BMI)

PMH:      hypertension
Meds:    lisinopril 40 mg daily, oxycodone 5 mg po q4 hours prn pain, ondansetron 4 mg po q4 hours prn

nausea

**Student Tasks:**

Take a palliative care history from a patient who has been told there are no more cancer treatments available for him/her. You have up to 20 minutes with the patient to gather the necessary information to form a palliative care plan for the patient. You do not need to do a physical exam.

**Communication Behavior Checklist for Student Observer**

**(also suitable for other assessors – SPs, faculty, etc.)**

**Complete the following checklist during the interview as you observe:**

| 1. Opens up dialogue to explore goals of care. | ( ) Yes | ( ) Partial | ( ) No |
| --- | --- | --- | --- |
| 2. Elicits patient’s understanding of disease and prognosis. | ( ) Yes | ( ) Partial | ( ) No |
| 3. Inquires about pain and pain management. | ( ) Yes | ( ) Partial | ( ) No |
| 4. Inquires about nausea or other common physical symptoms in patients with palliative care needs (e.g. weight loss, fatigue). | ( ) Yes | ( ) Partial | ( ) No |
| 5. Inquires in open-ended fashion about physical feelings (and/or noting low blood pressure). | ( ) Yes | ( ) Partial | ( ) No |
| 6. Explores presence of depression/anxiety. | ( ) Yes | ( ) Partial | ( ) No |
| 7. Explores presence of existential concerns/distress. | ( ) Yes | ( ) Partial | ( ) No |
| 8. Asks whether patient is using any complementary treatment. | ( ) Yes | ( ) Partial | ( ) No |
| 9. Inquires about living situation and social supports. | ( ) Yes | ( ) Partial | ( ) No |
| 10. Inquires about family or financial concerns. | ( ) Yes | ( ) Partial | ( ) No |
| 11. Inquires about health care proxy. | ( ) Yes | ( ) Partial | ( ) No |
| 12. Inquires about whether patient has a living will or end-of-life preferences. | ( ) Yes | ( ) Partial | ( ) No |
| 13. Inquires about spirituality or religion. | ( ) Yes | ( ) Partial | ( ) No |
| 14. Explores whether patient knows about hospice. | ( ) Yes | ( ) Partial | ( ) No |

**Modified Master Interview Rating Scale (MIRS) for Student Observer**

**(also suitable for other assessors – SPs, faculty, etc.)**

The full MIRS can be found in Supplement 1 of Baldwin JD, Cox J, Wu ZH, Kenny A, Angus S. Delivery and Measurement of High-Value Care in Standardized Patient Encounters. Journal of Graduate Medical Education. 2017;9:645-449. [https://doi.org/10.4300/JGME-D-17-00016.1](https://nam12.safelinks.protection.outlook.com/?url=https%3A%2F%2Fdoi.org%2F10.4300%2FJGME-D-17-00016.1&data=04%7C01%7Cjaideep.talwalkar%40yale.edu%7Cb3f1b75239754625b97308d8cecb8002%7Cdd8cbebb21394df8b4114e3e87abeb5c%7C0%7C0%7C637486720727321375%7CUnknown%7CTWFpbGZsb3d8eyJWIjoiMC4wLjAwMDAiLCJQIjoiV2luMzIiLCJBTiI6Ik1haWwiLCJXVCI6Mn0%3D%7C3000&sdata=JvGyuedfM5vMJOVISVQZh1SaegcnYwZzlZCp2cdfZbw%3D&reserved=0)

Items from MIRS used by Student Observers:

1. Opening

12. Questioning Skills – Lack of Jargon

14. Interactive Techniques

15. Verbal Faciliation Skills

16. Non-Verbal Facilitation Skills

17. Empathy and Acknowledging Patient Cues

22. Patient’s Education & Understanding

27. Encouragement of Questions

28. Closure

**Case script for Standardized Patient**

**Standardized Patient Name:** use your regular character’s name
**Actor:**

**Age:** 50-60
**Episode:** Palliative Care
**Workshop:** Advanced Communication Skills, Fourth Year Capstone Course
____________________________________________________________________________________

You are following up in your primary care office today but are meeting a new doctor.

You began to experience pain in your stomach and back and began to lose some weight about 8 months ago. After a couple of weeks you went to the doctor who sent you for blood work and X rays. The testing led to a diagnosis of cancer of the pancreas and you began treatment for it with chemotherapy 6 months ago. Two months into therapy, repeat scans showed that the cancer continued to grow so you were switched to another regimen. That therapy has not halted the spread of the cancer and your cancer doctor told you last week that there are no other treatments she can offer. Since the chemotherapy made you feel very sick, you are accepting of her advice. The main focus of your treatment now is on keeping you comfortable. You are fatigued, have lost your hair and 40 lbs. The oncologist sent you back to your primary care doctor to manage your symptoms and other medical problems and that is why you are here today.

At the moment you are taking lisinopril 40 mg for your high blood pressure (you have been on this medicine for over 10 years), oxycodone 5 mg as needed about twice a day for pain, and ondansetron (“Zofran") 4 mg once or twice a week for nausea. Your symptoms are well controlled and you know you could take more medicine if you were more uncomfortable. You have also begun to experience some light headedness when you stand up quickly. You sleep “okay,” but not great. You do not eat as much because your appetite has decreased.*

You worked as a teacher of theater at your local college and had been able to continue with some sick days. You found it challenging to go to work and the end of the school year was a relief. You have good health insurance through work. You are happily married and you are worried** about how your spouse will manage financially without the dual income. However, you know “it will be okay,” as your spouse is employed as a manager of the local grocery store and is able to take time off to care for you as needed. You have 2 adult children who are working and living independently. They know you are ill and are very sad about your declining health. Both are willing and able to help at home. You do have life insurance that will help some. Your home is paid for.

You are also pondering the question of why you are terminally ill so early in your life. You pray and meditate and that provides some comfort. On the other hand you have been doing practical things like making out a will and thinking about who will make health care decisions if you cannot. You are an active Roman Catholic who is very involved in the local church. You have also decided that you want to die a natural death when the time comes avoiding resuscitation, breathing and feeding tubes and your partner knows this.

You have used some of your family’s home remedies of taking bitter melon juice, but do not have other cultural beliefs about treating cancer. You have heard of hospice and are interested in having their support since you wish to remain at home to the extent that is possible. Your goals for the next few months are to spend time with your family and be spiritually ready for your death if there is no miracle.

You are not a smoker, do not drink alcohol, nor do you use drugs like marijuana. There is no family history of cancer.

* If the student focuses too much on your cancer treatments and medical symptoms, gently redirect them and let them know that you fully understand your treatment status (“I’m done with chemo… I know there are no more options… I’m here to talk about next steps.”)

** Ideally the student will provide a natural opening for you to express your emotions. Suitable windows could be inquiries about your family, mental health, or existential concerns, or more direct questions about your emotions. “Worry” about your partner’s well-being is your primary emotional concern, and thinking about this may make your character cry. If the student recognizes your emotion and demonstrates empathy, you should be able to collect yourself and move forward with the conversation. If the student is not effective in handling your emotion, the conversation will stall as you find it difficult to move beyond your worry, which could then escalate to other emotions (fear, isolation, etc.).

***MedEdPORTAL* Standardized Patient Case Development Tool**

Date: January 26, 2021

Primary Case Author: Yale School of Medicine Advanced Communication Skills Workshop group

Secondary Case Author: Not applicable

Standardized Patient Educator: Not applicable

Name of Case: Palliative Care

Name of educational and or assessment activity: Advanced Communication Skills Workshop

Patient Name: Character’s regular name

Chief Concern: Terminal pancreatic cancer

Most likely Diagnosis and Differential with rationale from history and/or physical exam: Not applicable

Challenge question:

You are a medicine intern in the primary care clinic. This patient has been coming to the clinic for 5 years for treatment of his/her hypertension, but you have not personally met the patient before.

The patient is a (insert actor’s age)-year-old man/woman who was diagnosed with pancreatic carcinoma 7 months ago. He/she has received 2 different chemotherapy drugs over 6 months and neither worked. He/she received the most recent chemotherapy 3 weeks ago, but the cancer has continued to spread. His/her oncologist has said there are no other treatment options for the cancer. You have read a report from the oncologist that states he/she knows the full extent of his/her disease and that no further cancer specific treatment is available.

Vital Signs:  T: 37.1C, P: 76, R: 16, BP 95/60, Pulse Ox: 99% on Room Air
Weight/Height/BMI: (insert actors’s weight/height/BMI)

PMH:      hypertension
Meds:    lisinopril 40 mg daily, oxycodone 5 mg po q4 hours prn pain, ondansetron 4 mg po q4 hours prn

nausea

Your task is to take a palliative care history from a patient who has been told there are no more cancer treatments available for him/her. You have up to 20 minutes with the patient to gather the necessary information to form a palliative care plan for the patient. You do not need to do a physical exam.

Domains: Check all that apply

- Professionalism
- Communication and Interpersonal skills
- Medical History
- Physical exam
- Shared Decision Making
- Patient Education
- Clinical Reasoning
- Documentation
- Handoff
- Presentation
- Other:

Type and level of learner: Senior medical student

Case Objectives: please list specific objectives for each of the domains you have checked above:

1. Interview a patient with terminal illness with the specific purpose of discussing elements in a palliative care history

2. Respond to emotions exhibited by a patient with verbal empathic statements

3. Respond to emotions exhibited by a patient with appropriate body language

| SETTING: outpatient, in patient, ED, home, nursing home, rehab, group etc. | Outpatient primary care office |
| --- | --- |
| PATIENT PROFILE: Information about the “patient” that helps select an SP and helps the learner get an understanding of them as a person. SP will know more information about the patient than learner will ever ask but allows SP to portray a fully developed patient personality. If none of the items below are particulars for the case please write “all may be used.” | |
| Age range | 50-60 |
| Religious/spiritual background | Roman Catholic |
| Sex (e.g., male, female, intersex, transwoman, transman) | All may be used |
| Sexual Orientation (e.g., heterosexual, lesbian, gay, bisexual, pansexual, queer, asexual) | All may be used |
| Gender expression (e.g., man, woman, gender queer) | All may be used |
| Race/ethnicity: | All may be used |
| Physical description (e.g., BMI, height range) | Patient is chronically ill and has lost 40 pounds in past 8 months. Patient has lost hair from chemotherapy (may wear a bandana or other head covering to demonstrate this if needed). |
| Physical limitations | All may be used |
| Patient appearance (e.g., disheveled, hospital gown, business casual, casual) | Casual |
| Moulage + location (e.g., none, bruises, scars, body piercing, tattoos) | None |
| Affect (e.g., pleasant, cooperative) | Initially matter of fact, expressing clear understanding of prognosis. Eventually will become emotional (see below for details) as interviewer inquires into personal and emotional contexts of patient’s story. |
| Family group (e.g., who is family, who they live with) | Happily married. Has 2 adult children who are working and living independently. They know you are ill and are very sad about your declining health. Both are willing and able to help at home. |
| Education | College graduate |
| Level of health literacy | Adequate; has good understanding of medical history and prognosis. |
| Employment, if any - present and past, noting any current stresses | Worked as a teacher of theater at a local college and had been able to continue with some sick days. Found it challenging to go to work and the end of the school year was a relief. |
| Home/homeless - type of dwelling, number of stories, owned or rented | Owns home with spouse; home is paid for. |
| Financial situation- any current stresses | You are worried about how your spouse will manage financially without the dual income once you are unable to work. However, you know “it will be okay,” as your spouse is employed as a manager of the local grocery store and is able to take time off to care for you as needed. |
| Insurance Status (e.g., un/under/insured, public/private, HMO/PPO) | You have good health insurance through work. You have life insurance that will help some with family finances when you die. |
| Habits (i.e., diet, exercise, caffeine, smoking, alcohol, drugs) | You are not a smoker, do not drink alcohol, nor do you use drugs like marijuana. |
| Activities (i.e., hobbies, sports, clubs, friends) | Very involved in the local church. |
| Typical day - what is the usual daily routine | All may be used, but in past few weeks feeling some light headedness when you stand up quickly which is limiting activity, in addition to general fatigue, and intermittent nausea and abdominal pain. |

| CASE INFORMATION | |
| --- | --- |
| Chief Concern: What the patient will say when greeted by the student. The patient’s primary reason for seeking medical care often stated in his/own words. | The patient knows that he/she has terminal pancreatic cancer and is being sent by oncologist back to the primary care doctor to manage symptoms and other medical problems. |
| Additional Concerns: Other, if any, concerns the patient has today (i.e., symptoms, requests, expectations, etc.) that will become part of set agenda. |  |
|  | |
| THE PATIENT STORY: The SP will be asked to tell their symptom story and the personal and emotion impact for each of their concerns. You will want to write this is the patient voice. The symptom story should be able to answer this question: “Tell me more about [chief concern/additional concern], starting at the beginning and bringing me up to now.”  The personal context should be able to answer questions concerning the broader personal/psychosocial context of symptoms, especially the patient beliefs/attributions.  The emotional context should be able to ask how are you doing with this, how does this make you feel, how has this affected you emotionally? IMPACT: How has this affected your life? How has this been for your family? | The patient is following up in the regular primary care office today but is meeting a new doctor.  You began to experience pain in your stomach and back and began to lose some weight about 8 months ago. After a couple of weeks you went to the doctor who sent you for blood work and X rays. The testing led to a diagnosis of cancer of the pancreas and you began treatment for it with chemotherapy 6 months ago. Two months into therapy, repeat scans showed that the cancer continued to grow so you were switched to another regimen. That therapy has not halted the spread of the cancer and your cancer doctor told you last week that there are no other treatments she can offer. Since the chemotherapy made you feel very sick, you are accepting of her advice. The main focus of your treatment now is on keeping you comfortable. You are fatigued, have lost your hair and 40 lbs. The oncologist sent you back to your primary care doctor to manage your symptoms and other medical problems and that is why you are here today.  At the moment you are taking lisinopril 40 mg for your high blood pressure (you have been on this medicine for over 10 years), oxycodone 5 mg as needed about twice a day for pain, and ondansetron (“Zofran") 4 mg once or twice a week for nausea. Your symptoms are well controlled and you know you could take more medicine if you were more uncomfortable. You have also begun to experience some light headedness when you stand up quickly. You sleep “okay,” but not great. You do not eat as much because your appetite has decreased.  You worked as a teacher of theater at your local college and had been able to continue with some sick days. You found it challenging to go to work and the end of the school year was a relief. You have good health insurance through work. You are happily married and you are worried about how your spouse will manage financially without the dual income. However, you know “it will be okay,” as your spouse is employed as a manager of the local grocery store and is able to take time off to care for you as needed. You have 2 adult children who are working and living independently. They know you are ill and are very sad about your declining health. Both are willing and able to help at home. You do have life insurance that will help some. Your home is paid for.  You are also pondering the question of why you are terminally ill so early in your life. You pray and meditate and that provides some comfort. On the other hand you have been doing practical things like making out a will and thinking about who will make health care decisions if you cannot. You are an active Roman Catholic who is very involved in the local church. You have also decided that you want to die a natural death when the time comes avoiding resuscitation, breathing and feeding tubes and your partner knows this.  You have used some of your family’s home remedies of taking bitter melon juice, but do not have other cultural beliefs about treating cancer. You have heard of hospice and are interested in having their support since you wish to remain at home to the extent that is possible. Your goals for the next few months are to spend time with your family and be spiritually ready for your death if there is no miracle.  You are not a smoker, do not drink alcohol, nor do you use drugs like marijuana. There is no family history of cancer.  If the student focuses too much on your cancer treatments and medical symptoms, gently redirect them and let them know that you fully understand your treatment status (“I’m done with chemo… I know there are no more options… I’m here to talk about next steps.”)  Ideally the student will provide a natural opening for you to express your emotions. Suitable windows could be inquiries about your family, mental health, or existential concerns, or more direct questions about your emotions. “Worry” about your partner’s well-being is your primary emotional concern, and thinking about this may make your character cry. If the student recognizes your emotion and demonstrates empathy, you should be able to collect yourself and move forward with the conversation. If the student is not effective in handling your emotion, the conversation will stall as you find it difficult to move beyond your worry, which could then escalate to other emotions (fear, isolation, etc.). |
| HISTORY OF PRESENT ILLNESS: Although some of the HPI will be given in the patient’s symptom story, the learners will expand the story during the direct question section. Below describe the detailed history, usually about the chief concern, which the student must develop in order to make a useful assessment of the problem: | |
|  | |
| Onset (when; gradual or sudden) | Worsening over past 8 months |
| Setting (what was going on or where was patient when symptoms first noticed?) | The patient began to experience stomach and back pain with weight loss about 8 months ago. |
| Duration (how long) | 8 months |
| Time relationships (frequency, constant or intermittent) | Intermittent |
| Location | Abdomen and back |
| Radiation | Should not be the focus of the history, but pain goes to mid-back |
| Quality | Should not be the focus of the history, but pain is crampy |
| Amount | Daily |
| Aggravated by what | Occurs at random |
| Relieved by what | Passes on its own or with oxycodone |
| Associated with what | Nausea, though hard to tell if nausea is from chemotherapy |
| Attitude (what does the patient think is the problem, and how does he/she feel about it) | Patient is pondering the question of why he/she is terminally ill so early in life. Prays and meditates and that provides some comfort. |
| Overall course | See above |
| REVIEW OF SYSTEMS: Significant positives and negatives | |
|  | See above |
|  |  |
|  |  |
|  |  |
|  | |
| Past medical history |  |
| Medication allergies (Name and reaction) | All may be used, but not relevant to case |
| Environmental allergies (Name and reaction) | All may be used, but not relevant to case |
| Illnesses | Pancreatic cancer, Hypertension |
| Vaccinations | All may be used, but not relevant to case |
| Surgeries | None |
| Accidents/ injuries/ trauma | All may be used, but not relevant to case |
| Hospitalization | All may be used, but not relevant to case |
|  | |
| Inclusive sexual and reproductive history | |
| Sexual practices  Sexual partners  Protection: Use of safer sex practices  Use of birth control if appropriate  Risk of intimate partner violence | All may be used, but not relevant to case |
| Ob/GYN HISTORY | Age of onset of menses --- Not relevant to case  Age of menopause  Number of pregnancies  Number of live births  Number of miscarriages  Number of abortions |
| Medications | Prescription/dose/reason  lisinopril 40 mg daily for hypertension;, oxycodone 5 mg po q4 hours prn pain; ondansetron 4 mg po q4 hours prn nausea  Over the counter/dose/reason  Herbs/supplements/dose/reason  Other: |
| Immunizations | - Tetanus --- Not relevant to case - Flu - Hepatitis - Pneumovax - HPV - Other |
| Tobacco products:   - Cigarettes - Cigar - Pipe - Chew - E-cigarettes | - Never - Past- year started/year quit - Current   - Quantity   - # of years |
| Alcohol   - Beer - Wine - Liquor - Other | - Never - Past- year started/year quit - Current   - Quantity   - # of years |
| Drugs   - Weed - Cocaine - Heroin - Meth - Other - IV - Inhalants - Other | - Never - Past- year started/year quit - Current   - Quantity - # of years |
| Diet (describe) | You do not eat as much because your appetite has decreased |
| Exercise (describe) | Not relevant to case |
| List any other important social history or information important to this case | See above |
| Family history |  |
| Mother, Father, Siblings, Grandparents, and other significant findings. | Not relevant to case |
|  |  |
| Physical Exam- List exam maneuvers expected for this case and any abnormal findings that SP will simulate. (tenderness, hyper-hypo reflex, rebound, weakness etc. )  No physical exam as part of this case. | |
| PHYSICAL EXAM FINDINGS |  |
| 1. Written in layman’s terms | N/A |
| 1. General appearance- affect, appearance, position of patient at opening (i.e. sitting, laying down, holding abdomen etc.) | Appears comfortable (currently no pain or nausea) though with some generalized psychomotor slowing. |
| 1. Vital signs | T: 37.1C, P: 76, R: 16, BP 95/60, Pulse Ox: 99% on Room Air |
| 1. Specific findings and affect | See description of emotional reactions above. |
| 1. Response to certain physical movements | none |
|  |  |
| DIAGNOSIS AND DIFFERENTIAL |  |
| Diagnosis with support from positive and negative history and PE findings | Not relevant to case |
| Differential with support from positive and negative history and PE findings | Not relevant to case |
|  |  |
| MANAGEMENT OR DIAGNOSTIC PLAN | This case is focused on taking a palliative care history. The interviewer should cover the domains listed on the Communication Behavior Checklist, including inquiries about symptom control, social supports, finances, and end-of-life preferences. Additionally, the interviewer should conisider iatrogenic contributions to the patient’s symptoms (anti-hypertensive medicine). Recognizing and addressing the patient’s emotional response to terminal illness is also critical to a successful interview. |
|  |  |
| PROFESSIONALISM ISSUES OR CHALLENGES: | Through inquiry into the patient’s understanding of the medical situation, the interviewer should recognize early in the visit that the focus should be on domains of palliative care rather than a recap of the past 8 months of medical history. The interviewer must take care to address the patient’s emotion in order to move to a successful discussion about palliative care. |
