# Supplementary material for: An Advanced Communication Skills Workshop Using Standardized Patients for Senior Medical Students
Source: MedEdPORTAL. 2021 May 27;17:11163. doi: 10.15766/mep_2374-8265.11163 (PMC8155077; doi:10.15766/mep_2374-8265.11163)
Supplement: Supplementary file 1 — Schedule & Logistics.xlsxStrong Emotion Case Materials.docxGoals of Care Case Materials.docxError Disclosure Case Materials.docxPalliative Care Case Materials.docxStudent Instructions.docxPostsession Survey.docxFaculty Debrief Guide.docx [file mep_2374-8265.11163-s001.zip › F. Student Instructions.docx]

Appendix F

**Advanced Communications Skills Workshop**

**Session Outline**

1. Students are assigned to a group of 3 (example below) and instructed to report to their exam room 5 minutes before the arrival time listed on the schedule for their wave. (Note: Each interview must begin on time whether all students have arrived or not. Students who arrive late must wait in the hall until completion of the first interview.)

| **Time** |  | **Room 1** |
| --- | --- | --- |
| **7:55 am - 10:10 am** | 1^st^ Interview (8:00-8:30) | Depa Billaba |
|  | 2^nd^ Interview (8:35-9:05) | Lott Dod |
|  | 3^rd^ Interview 3 (9:10-9:40) | Rush Clovis |
|  | 9:40-10:10 | Debrief in Conference Room |

1. Outside in the hallway the **interviewing student** (Depa) is given 1-2 minutes to review the opening scenario of the case (there will be a holder on the exam room door with the case attached).
2. Inside of the exam room the two **observing** students (Lott and Rush) will look at the schedule posted to the door to see which case they are observing (example below). In this example, it would be the Topic A case. They will pull up the corresponding case document in the course management system which has the opening scenario and instructions on first page and the history checklist on the second page. There are also copies of laminated MIRS checklists in each room.

| **Room 1** | | |
| --- | --- | --- |
| **Time** | **Student** | **Case** |
| 1^st^ Interview (8:00-8:30) | Depa Billaba | Topic A |
| 2^nd^ Interview (8:35-9:05) | Lott Dod | Topic B |
| 3^rd^ Interview (9:10-9:40) | Rush Clovis | Topic C |

1. At 8 am, Depa enters the exam room and conducts an interview of the standardized patient. Students have 20 minutes maximum to complete the interview, but may finish sooner. There will be a 5-minute warning knock at 8:15 am to keep the interviewer on pace.
2. Our observing students, Lott and Rush, will observe Depa’s interview and complete a history and MIRS checklist to use in providing feedback to Depa.
3. At the end of the 20 minute interview, 8:20 am, Depa will take a 1-2 mins to complete a self-assessment checklist (found on back of the opening scenario) while Lott and Rush gather their thoughts.
4. By 8:22 am, Lott and Rush begin to provide feedback to Depa based on the checklist items. Feedback wraps up by 8:30 am.
5. At 8:30 am, Lott now becomes the interviewer and comes out into the hallway for 5 minutes to review the door note. Depa and Rush are now the observers and pull up the checklist from the course management system for Lott’s assigned case, which is Topic B. Lott begins the interview at 8:35 am.
6. We proceed as above. Rush becomes the third interviewer and comes out into the hallway to review the door note at 9:05 am.
7. At 9:40 am, after all three students have conducted interviews, they will proceed along with the other students assigned to the first wave to the conference room for a faculty debrief of the cases. Students can ask questions about the cases and checklists, and discuss emotions, challenges, and strategies for success.
