# Supplementary material for: An Advanced Communication Skills Workshop Using Standardized Patients for Senior Medical Students
Source: MedEdPORTAL. 2021 May 27;17:11163. doi: 10.15766/mep_2374-8265.11163 (PMC8155077; doi:10.15766/mep_2374-8265.11163)
Supplement: Supplementary file 1 — Schedule & Logistics.xlsxStrong Emotion Case Materials.docxGoals of Care Case Materials.docxError Disclosure Case Materials.docxPalliative Care Case Materials.docxStudent Instructions.docxPostsession Survey.docxFaculty Debrief Guide.docx [file mep_2374-8265.11163-s001.zip › G. Postsession survey.docx]

**Advanced Communications Skills Workshop**

**Post-Session Survey**

Today’s Advanced Communication Skills Workshop is a new addition to the medical school curriculum. Please share your thoughts with us on your experience using this 5-minute survey. The feedback you provide here is anonymous, voluntary, and optional. It will directly inform changes and improvements to the session for future classes. We greatly appreciate your time and attention!

For which case did you perform the role of Interviewer?

- Strong Emotion (unexpected ileostomy)
- Disclosure of Error (perforated ulcer)
- Goals of Care (father in ICU)
- Palliative Care (terminal pancreatic cancer)

Items 1-3 – Use the scale to answer the following items.

|  | **Excellent** | **Above Average** | **Average** | **Below Average** | **Poor** |
| --- | --- | --- | --- | --- | --- |
| Overall, the educational value of the session where I was the Interviewer was: | O | O | O | O | O |
| How valuable was the feedback you received from your peers about your interview? | O | O | O | O | O |
| How valuable was the self-reflection checklist you completed when you were the Interviewer? | O | O | O | O | O |

For which two cases did you perform the role of Observer?

- Strong Emotion (unexpected ileostomy)
- Disclosure of Error (perforated ulcer)
- Goals of Care (father in ICU)
- Palliative Care (terminal pancreatic cancer)

Items 4-5 – How beneficial was your participation as the Observer in terms of:

|  | **Excellent** | **Above Average** | **Average** | **Below Average** | **Poor** |
| --- | --- | --- | --- | --- | --- |
| Improving your knowledge about the topic? | O | O | O | O | O |
| Practicing giving feedback to peers? | O | O | O | O | O |

Free Response – What are your thoughts about the peer-feedback process in today’s session? What was your experience giving and receiving feedback? How useful were the checklists to help you formulate your feedback?

____________________________________________________________________________________
