# Supplementary material for: An Advanced Communication Skills Workshop Using Standardized Patients for Senior Medical Students
Source: MedEdPORTAL. 2021 May 27;17:11163. doi: 10.15766/mep_2374-8265.11163 (PMC8155077; doi:10.15766/mep_2374-8265.11163)
Supplement: Supplementary file 1 — Schedule & Logistics.xlsxStrong Emotion Case Materials.docxGoals of Care Case Materials.docxError Disclosure Case Materials.docxPalliative Care Case Materials.docxStudent Instructions.docxPostsession Survey.docxFaculty Debrief Guide.docx [file mep_2374-8265.11163-s001.zip › H. Faculty Debrief Guide.docx]

Appendix H

Advanced Communication Skills Workshop

Faculty Debrief Guide

As part of the Capstone Course, 4^th^ year students will rotate through 3 of the following standardized patient practice stations:

- Goals of Care – a discussion with a family member of a dying patient in the ICU
- Disclosure of Error – a discussion with a patient after the doctor neglected to follow up on an abnormal Xray result
- Strong Emotion – a discussion with a patient who is angry about a known possible surgical complication
- Palliative Care – a discussion with a patient with a terminal cancer diagnosis about palliative care options.

Students will interview the patient at one station, and observe classmates conducting the interview at two stations. Students will give and receive feedback from classmates based on checklists created by faculty who are content experts. Students will also complete self-assessment checklists.

After students have completed their 3 stations, we will ask them to meet for a 30 minute debrief with faculty. During the debrief, consider the following discussion points:

- How had the curriculum to this point prepared you for these conversations?
- Have you seen similar discussions with real patients? Who led these conversations? How were the approaches you’ve witnessed similar or different than what we wanted you to do today?
- How did you find the observer role? Did the checklists keep you engaged, help you internalize the content, and give you points for discussion for feedback?
- How was it receiving feedback from your classmates? (If students express concern about lack of direct feedback from faculty, you might tell them that our study of this session in the past indicated that students really liked the peer feedback model. Peer evaluation was similar to faculty evaluation, self-evaluation, and SP evaluation in our study.)
- What questions do you have about the checklists themselves? How ready do you feel to lead conversations on topics like this in a few months when you are interns?
- What other advanced communication skills topics would you have liked to practice?

Please share constructive comments and suggestions with the course director. We will compile your comments and share them with the working group in charge of the content for the Advanced Communication Skills Workshop.

Thanks!
